# ORCA-SPY enables killer whale sound source simulation, detection, classification and localization using an integrated deep learning-based segmentation

**DOI:** 10.1038/s41598-023-38132-7

**Published:** 2023-07-10

**Authors:** Christopher Hauer, Elmar Nöth, Alexander Barnhill, Andreas Maier, Julius Guthunz, Heribert Hofer, Rachael Xi Cheng, Volker Barth, Christian Bergler

**Affiliations:** 1 Pattern Recognition Lab, Department of Computer Science, Friedrich-Alexander-Universität Erlangen-Nürnberg, Martensstr. 3, 91058 Erlangen, Germany; 2 Leibniz Institute for Zoo and Wildlife Research (IZW), Alfred-Kowalke-Straße 17, 10315 Berlin, Germany; 3 Department of Veterinary Medicine, Freie Universität Berlin, 14195 Berlin, Germany; 4 Department of Biology, Chemistry, Pharmacy, Freie Universität Berlin, 14195 Berlin, Germany; 5 Anthro-Media, Nansenstr. 19, 12047 Berlin, Germany; 6 Universität des Saarlandes, 66123 Saarbrücken, Germany

**Keywords:** Marine biology, Machine learning, Software

## Abstract

Acoustic identification of vocalizing individuals opens up new and deeper insights into animal communications, such as individual-/group-specific dialects, turn-taking events, and dialogs. However, establishing an association between an individual animal and its emitted signal is usually non-trivial, especially for animals underwater. Consequently, a collection of marine species-, array-, and position-specific ground truth localization data is extremely challenging, which strongly limits possibilities to evaluate localization methods beforehand or at all. This study presents ORCA-SPY, a fully-automated sound source simulation, classification and localization framework for passive killer whale (*Orcinus orca*) acoustic monitoring that is embedded into PAMGuard, a widely used bioacoustic software toolkit. ORCA-SPY enables array- and position-specific multichannel audio stream generation to simulate real-world ground truth killer whale localization data and provides a hybrid sound source identification approach integrating ANIMAL-SPOT, a state-of-the-art deep learning-based orca detection network, followed by downstream Time-Difference-Of-Arrival localization. ORCA-SPY was evaluated on simulated multichannel underwater audio streams including various killer whale vocalization events within a large-scale experimental setup benefiting from previous real-world fieldwork experience. Across all 58,320 embedded vocalizing killer whale events, subject to various hydrophone array geometries, call types, distances, and noise conditions responsible for a signal-to-noise ratio varying from $-14.2$ dB to 3 dB, a detection rate of 94.0 % was achieved with an average localization error of 7.01$^\circ$. ORCA-SPY was field-tested on Lake Stechlin in Brandenburg Germany under laboratory conditions with a focus on localization. During the field test, 3889 localization events were observed with an average error of 29.19$^\circ$ and a median error of 17.54$^\circ$. ORCA-SPY was deployed successfully during the DeepAL fieldwork 2022 expedition (DLFW22) in Northern British Columbia, with a mean average error of 20.01$^\circ$ and a median error of 11.01$^\circ$ across 503 localization events. ORCA-SPY is an open-source and publicly available software framework, which can be adapted to various recording conditions as well as animal species.

## Introduction

Acoustic monitoring has a wide range of potential applications, including important ecological metrics (population trends and animal density) and providing detailed inferences on animal behavior^1, 2^. One of the most well-studied and charismatic toothed whale species is the killer whale (*Orcinus orca*)^3–13^. Killer whales live in stable matrilineal groups, and their highly social nature is reflected in a frequent and diverse vocal repertoire^6, 13, 14^. They are thus an ideal candidate species for acoustic monitoring. Yet the analysis of individual-specific/group-dependent vocalizations (speaker identification, group-specific dialects) does not suffice for context-dependent communication, as individual vocalizations could not yet be assigned to specific animal behaviors^15^. As such, turn-taking events (dialogs, conversations) should be examined as well, in order to gain deeper insights into killer whale communication. This requires robust machine-aided segmentation and subsequent localization techniques.

Nowadays, machine (deep) learning approaches are increasingly applied to segment/detect and classify animal-specific vocal activities on various taxonomic levels within noise-heavy bioacoustic data, such as bird species/call type detection and classification^16, 17^, right whale (*Eubalaena glacialis*) signal identification^18^, fin whale (*Balaenoptera physalus*) song note detection^19, 20^, whistle detection/extraction for toothed whales (*Odontoceti*)^21, 22^, sperm whale (*Physeter macrocephalus*) echolocation click detection as well as individual, coda type, and dialect classification^23^, ultrasonic bat search-phase echolocation recognition^24^, odontocetes echolocation click versus non-click event detection^25^, and koala (*Phascolarctos cinereus*) vocal activity identification^26^. Such machine-based segmentation/detection and classification algorithms act as pre-processing and filtering techniques with respect to a subsequent sound source localization algorithm, which then exclusively determines the signal positions of interest.

The same sound source localization algorithms conducted in the domain of human speech^27–30^ are also increasingly performed in bioacoustic to locate and identify certain individuals. Different recording setups and array geometries, in combination with distinct localization techniques, were applied to localize and track animal species, such as Difference-of-Arrival (DOA)-based analysis for bird localization in a native New Zealand forest using a linear microphone array^31^, or applying the MUSIC algorithm for bird localization utilizing a tripod with mounted microphones^32^. In addition, Time-Difference-Of-Arrival (TDOA)-based methods were applied in passive acoustic monitoring scenarios for fin whale (*Balaenoptera physalus*) tracking in the Gulf of Alaska^33^, Gulf of California^34^, and Southern California Offshore Range^35^. Plenty of TDOA-based animal localizations have also been conducted at the U.S. Navy’s Pacific Missile Range Facility (PMRF) in the northwest of the island of Kauai in the Hawaiian islands using a large-scale passive acoustic monitoring network in order to localize and track minke whales *(Balaenoptera acutorostrata)*^36^, fin whales (*Balaenoptera physalus*)^37^, humpback whales (*Megaptera novaeangliae*)^38, 39^, and bryde’s whales (*Balaenoptera brydei*)^40^. Furthermore, research has been conducted regarding real-time detection, localization, and tracking of Antarctic blue whales (*Balaenoptera musculus*) utilizing PAMGuard together with Directional Frequency Analysis And Recording (DIFAR) sonobuoys and a final beamforming algorithm^41^. Additional research in which sperm whales (*Physeter macrocephalus*) were localized within the waters of the Gulf of Maine was performed using a towed hydrophone array and the application of time-domain beamforming and moving array triangulation^42^. Moreover, harbor porpoise (*Phocoena phocoena*) localization in turbulent tidal waters was conducted, in which the received TDOAs from a large aperture vertical hydrophone array were analyzed by applying a Markov chain Monte Carlo (MCMC) method^43^. Monitoring and localization of cetaceans around wind turbines using a system of 12 hydrophone units was performed by maximizing the log-likelihood function of the possible locations using a Simplex algorithm^44^.

Applying sound source localization algorithms, in combination with stationary and/or moving hydrophone arrays, enable the possibility to separate animal- and individual-specific vocalization patterns. The majority of marine localization algorithms deal with passive and stationary recording environments. Although acoustic tracking can be realized, there is no chance of simultaneous behavioral observation and/or visual identification. In the case of an active approach, e.g., using a research vessel, there exists the possibility of acoustic localization and additional visual photo-identification^8^ (photo-ID), alongside the opportunity to document additional behavioral observations and map them to the respective killer whale-specific acoustic events. Those obtained data repositories, including killer whale individual-specific vocalization and associated behavioral patterns, provide new and deeper insights into animal understanding. It requires a fully-automated and hybrid machine-based acoustic identification procedure consisting of signal detection and localization in order to collect such highly valuable individual-based acoustic data archives.

However, in both cases, a variety of challenges has to be considered within such localization-based data acquisition scenarios. Before a sound source can be localized, the corresponding signal of interest has to be identified. Detection accuracy and algorithmic specifications, required for machine-based segmentation, are very much dependent on the following factors:

1. the proportion between bioacoustic signals and environmental noise,
2. the mixture of signals from different species,
3. the degree of overlapping vocalization events,
4. the recording setup and environment,
5. the noise conditions, and
6. real-time versus offline processing.

The localization accuracy is strongly influenced by the chosen hydrophone array geometry, which in turn highly depends on the properties of the sound of interest (e.g., vocalization duration and spectral frequency ranges), as well as recording conditions and noise characteristics. Given this multitude of potential problems and challenges with respect to a sound source detection and localization, it is necessary to provide an acoustic localization framework that is not just capable of robustly detecting and localizing killer whale individuals in the field, but also provides a simulation framework in order to verify various recording setups, detection, and localization configurations, all together being as close as possible to real-world scenarios.

In order to handle all previously mentioned challenges and address the problem of killer whale localization, this work presents ORCA-SPY, to the best of the authors’ knowledge the first study introducing an automated acoustic sound source simulation and real-time localization framework for arbitrary microphone arrays. The framework, consisting of a hybrid approach between deep-learning-based bioacoustic event segmentation and subsequent localization, was embedded into PAMGuard^45^, which is a widely used bioacoustic software. ORCA-SPY integrates and combines a state-of-the-art deep-learning-based sound segmentation module, entitled ANIMAL-SPOT^46^, together with PAMGuard’s^45^ internal TDOA-based localization plugin. ANIMAL-SPOT^46^ is a continuation of the in previous work introduced ORCA-SPOT^47^. ANIMAL-SPOT can be adapted to arbitrary animal vocalizations and was shown to work for 10 different species ranging from Atlantic cod (gadus morhua) to bats (Pygmy pipstrelle, Pipistrellus pygmaeus). The ORCA-SPY and ANIMAL-SPOT source code is publicly available here^48^. ORCA-SPY’s simulation framework can be used to estimate the accuracy of arbitrary recording array constellations with bioacoustic target signals before field deployment. The structure of the paper is summarized as follows:

1. Methodology describes a detailed overview of the corresponding methodologies employed within this work. Most importantly, the ANIMAL-SPOT Network, the PAMGuard software and the subject of the paper, the combined tool chain ORCA-SPY.
2. Experiments describes the scenarios performed on the simulated experimental framework, the Lake Stechlin^49^ experiment in Northern Germany and the DeepAL fieldwork 2022 expedition (DLFW22) in Northern British Columbia.
3. The results of the experiments that are taken as basis for the following.
4. The discussion of the findings of the experiments.
5. Lastly, the conclusion and future work will give an outlook on how to possibly improve the tool chain, as well as on what to possibly come in the future.

## Methodology

It is explicitly mentioned that no animals were directly involved in this study. The data material utilized for simulation purposes is based on data samples originating from the Orchive^9, 50, 51^, which is accessible here^50^.

### ANIMAL-SPOT

For our experiments, we used a ResNet18-based^52^ Convolutional Neural Network (CNN) entitled ANIMAL-SPOT^46^ a continuation of ORCA-SPOT, which was adapted to work with PAMGuard since version 2.02.01^53^. ANIMAL-SPOT enables binary segmentation of target signals, and was trained and tested in a supervised manner, based on a labeled data corpus including 17,104 orcas and 44,323 noise excerpts. The data corpus incorporates samples of:

1. the Orchive^9, 50, 51^, one of the largest animal-specific bioacoustic data repositories, which was recorded over 25 years (1985–2010) by using a stationary hydrophone network in northern British Columbia
2. the 2017/2018 fieldwork expedition (DeepAL17 and DeepAL18) data archive, containing additional orca data material, collected via a 15-m research trimaran utilizing hull-mounted hydrophones and a custom-made towed array^46^.

ANIMAL-SPOT performs binary classification on pre-processed, augmented, transformed, and 0/1-dB-normalized power spectrograms, leading to a final network input shape of 256$\times$128 (256 frequency bins, representing 800 Hz to 10 kHz, as well as 128 time frames)^46^. To segment unseen recordings with respect to orca sound activities and environmental background noise ANIMAL-SPOT applies a sliding window approach, by utilizing a given window size, step size, and network confidence threshold, leading to a frame-wise classification output. ANIMAL-SPOT only decides whether a frame contains vocal killer whale activity or not, without distinguishing between various call types, as well as the number of vocalization activities, such as single calls, multiple calls, and/or overlapping vocalization parts. After ANIMAL-SPOT additional call-type classification is also possible to better target specific groups but the current approach within the scope of this work had no need for specific animal calltype target groups. ANIMAL-SPOT has a mean detection accuracy of 97.9%^46^. It is more robust towards noise interferences in comparison to other detection methods such as power threshold detectors. The network, in combination with a mid-range NVIDIA GTX 1050 Graphical Processing Unit (GPU), achieved a factor of 25 times faster than real-time^46^.

This real-time factor is necessary for in field research where one might be bound to weaker mobile hardware, such as laptops. Due to the power restrictions on research vessels and the presence of other computationally heavy applications that must run in parallel, the ANIMAL-SPOT ResNet18-based architecture was designed to not be too computationally heavy and to compute a sliding window in less than approximately half the time frame of a window size (i.e. take less than 1 s for a 2 s window) even without GPU acceleration.

### PAMGuard

Passive Acoustic Monitoring Guardianship (PAMGuard) is a set of tools for soundscape analysis, detection, classification, and localization of soniferous species^45^. It is primarily used to study cetaceans in the marine environment, providing users with a suite of no-code visualization, data management, and automated analysis tools^45^. While most of PAMGuard was designed towards marine environments, the physical model also allows for land-based observation of soniferous species, such as bats or birds. PAMGuard is based around a modular structure, allowing operators to tailor their setup depending on the acoustic workflow required. Modules are independent and thus, as new modules are created, they can be integrated without changing PAMGuard’s core code and take advantage of existing upstream and downstream modules^45^. Due to reasons of space and complexity, this work will focus hereinafter exclusively on the PAMGuard plugins utilized in the context of this study. However, more detailed information about all available PAMGuard modules, and functionalities, together with a user guide, are available on the official PAMGuard website^54^. Within the scope of this study, the following PAMGuard modules were an indispensable building block of the entire orca sound localization environment^45, 54^:

1. Sound acquisition,
2. Sound recorder,
3. the raw deep learning classifier, and
4. the Bearing calculator, applying the embedded Time-Difference-Of-Arrival (TDOA) algorithm.

#### Sound acquisition

The sound acquisition module acquires raw sound data from different sources, e.g., data acquisition devices, servers, and sound files. The sound acquisition module contains a simulated acquisition (SimSAcq) option which allows users to simulate a source at a known location, source level intensity, and sound type. The sound type can be chosen from 13 built-in sound types (e.g., tonal sound, impulsive sound, porpoise click and chirps). The received sound data will then contain the simulated source sounds, along with the appropriate attenuation due to propagation and appropriate time offset if multiple distributed sensors are being used.

#### Sound recorder

The sound recorder captures acoustic data from a raw data source. During real-time operation, it is usually used to save full bandwidth or decimated sound files from the data acquisition system. In this context, the sound recorder was used to save the simulated sound data for additional analysis.

#### The FFT spectrogram engine

The FFT spectrogram engine provides basic spectrogram functionality, converting raw sound data into a spectrogram with a user-defined FFT length, hop size, and windowing function. The module also contains several optional noise reduction processes, such as click removal, which are detailed here^55^.

#### The raw Deep Learning Classifier

The raw Deep Learning Classifier (rDLC) module is part of the current 2.02 (Beta)-version of PAMGuard and performs deep learning-based target versus noise segmentation on a single channel. It provides corresponding detection events to downstream models in case of valid detection (network confidence larger than a given threshold). The raw Deep Learning Classifier is compatible with generic PyTorch generated models, but was developed with the ANIMAL-SPOT architecture in mind. The module contains the pre-processing algorithms to transform raw audio data into network accepted audio signals, such as segmented 0/1-dB-normalized power spectrograms.

#### Time-Difference-Of-Arrival (TDOA) localization

TDOA-based bearing localization, also known as hyperbolic bearing localization, locates the bearing of an unknown sound source (e.g., killer whale) using multiple distributed time-synchronized receivers (e.g., hydrophones) by measuring the TDOA between pairs of hydrophones, comparing one reference hydrophone against all others^56^. Differences in arrival times between pairs of hydrophones are used to approximate potential bearings of the sound source being modeled in the forms of hyperbolas^56, 57^. By cross-correlating the TDOA between all hydrophone pairs, the most-likely bearing target can be calculated from the potential bearings.

#### Bearing calculator

The bearing calculator is a generic localization module that accepts a variety of data types from upstream modules, including continuous raw sound data and detection/classification data. It utilizes a number of TDOA and beam-forming algorithms. In this study, ANIMAL-SPOT^46^ was set as the upstream module and used the standard TDOA grid search algorithm. This performs a spherical grid search around the hydrophone array to locate the most likely 3D bearing (horizontal −180$^\circ$ to 180$^\circ$ and vertical −90$^\circ$ to 90$^\circ$) for a set of received TDOA values. The angular resolution was set to 1$^\circ$ during the data validation.

### ORCA-SPY

The sound source simulation framework from ORCA-SPY is a multi-module-based approach integrated into PAMGuard, which is built upon a sequentially ordered plugin pipeline. The SimSAcq module was utilized to create multichannel audio streams. The SimSAcq module attenuates and temporally shifts a source file based on a near field spherical geometrical propagation model, the respective hydrophone constellation, the source position w.r.t the hydrophone constellation and the speed of sound underwater. The recordings contained vocalizing killer whale individuals with distinct call types and interfering boat noise at known locations and source intensity levels. By default, SimSAcq was not capable of simulating orca vocalizations, which required the PAMTRAIN code extension^58^. All simulated data recordings were stored as multichannel wave files using the sound recorder module. The rDLC module obtains the raw sound data from the Sound Acquisition module, either from a SimSAcq simulation, a previously stored multichannel recordings wave file, or a physical sound card in the field. The segmented and pre-processed data is classified by an ANIMAL-SPOT model, and the detection events are feed-forward to the bearing calculator module for localization. The results of the bearing calculator can also be displayed on a map.

## Experiments

**Figure 1:**
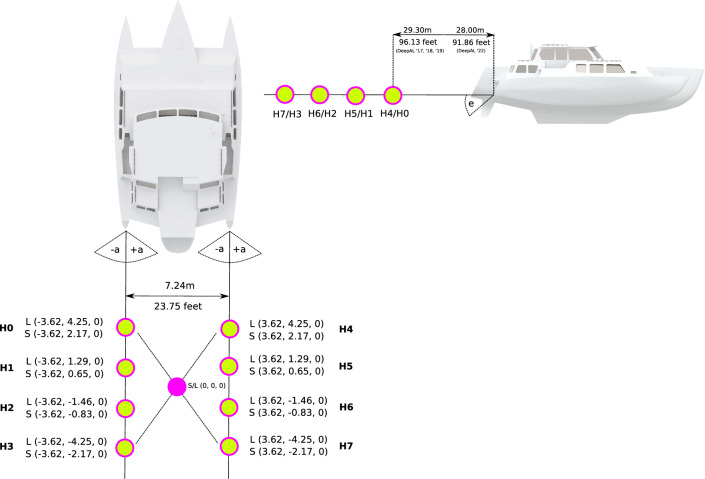
A depiction of the towed hydrophone Streamers from previous fieldwork expeditions^46^. For each hydrophone array, the (x, y, z)-coordinates with respect to the array origin are provided in Fig. 2. $\pm a$ and *e* illustrate the azimuth and elevation angle of the array. The arrays were deployed 29.3 m behind the vessel during prior field trips, during the DLFW22 expedition the new DeepAL array was deployed only 28 m behind the vessel.

In this study, the simulated experiments ((EXP-1) through (EXP-5)) were exclusively based on simulated localization data as realistic multichannel underwater audio streams, modelled after a variety of real-world situations from previous fieldwork expeditions^46^. A detailed analysis of the simulated scenarios can be found in Supplementary section Simulated Experiments. The DeepAL field experiment (EXP-6) was conducted during a field test of a new towed hydrophone array for future scientific expeditions under laboratory conditions at Lake Stechlin^49^ in northern Germany. The new towed hydrophone array was deployed during the DeepAL fieldwork 2022 expedition (DLFW22) in Northern British Columbia (EXP-7) for 3 weeks (Fig. 1).

### Hydrophone array composition

**Figure 2:**
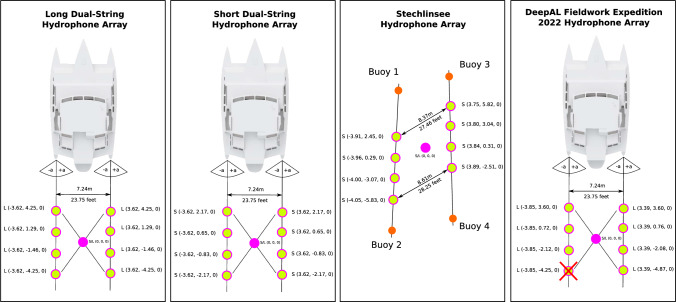
Depictions of the four hydrophone arrays utilized in this study. The x-, y- and z-coordinates display the distance of a hydrophone to the geometrical array center in meters. The geometrical center of an array also depicts the origin of the coordinate system.

Within the scope of this study, four different hydrophone array geometries were utilized, depicted in Fig. 2. The simulated long array (L) and short array (S) were inspired by the recording setups of previously conducted fieldwork expeditions^46^. The hydrophones of the short array (S) are approximately 1.5 m apart, whereas the long array (L) shows a distance of roughly 2.8 m. The first hydrophones (H0, H4) are 29.3 m during the 2019 expedition and 28 m during the 2022 expedition behind the end of the trimaran.

The Lake Stechlin Array was reconstructed from the GPS positions and in-field measurements of the new hydrophone array assembled during the lake Stechlin field experiment.

The streamers were attached onto two 10 m long rods to ensure that the streamer would stay straight and parallel during the recording sessions. By utilizing the 20 m long footbridge in Fig. 6, the assembled array was anchored 60 m away from shore at a depth of one meter below sea level using stone anchors and buoys. During the deployment, the distance between the first two buoys of the streamers and the last two buoys were measured to be 8.37 m and 8.60 m respectively. With uncertainty of about 10 cm due to water flow, the overall error to the parallelism of the streamers is 1.9$^\circ$. The DeepAL2022 Array was deployed during the DLFW22 expedition. The last hydrophone of the left streamer was damaged and had no outgoing signal before deployment. As such, the array was deployed utilizing the remaining seven hydrophones.

### Summary of simulated scenarios

A detailed summary of the simulated scenarios can be found in Supplementary section Simulated Experiments. Overall, five scenarios, in descending order of their complexity, were simulated utilizing the SimSAcq module, named (EXP-1) through (EXP-5). Both the short and long arrays were used in (EXP-1), (EXP-2) and (EXP-3), while (EXP-4) and (EXP-5) only used the long array.

1. In (EXP-1) both towed hydrophone arrays were evaluated using PAMGuard’s built-in 2 ms-long chirp-signal, which allows the best possible performance of PAMGuard’s TDOA bearing calculator while assuming ideal experimental conditions^44^.
2. (EXP-2) verifies the impact of various orca call type structures concerning localization accuracy. Nine call types (see Fig. 3) were utilized in combination with no added background noise, as well as interfering boat noise, resulting in SNR values between $+$ 3 dB and −4 dB.

**Figure 3:**
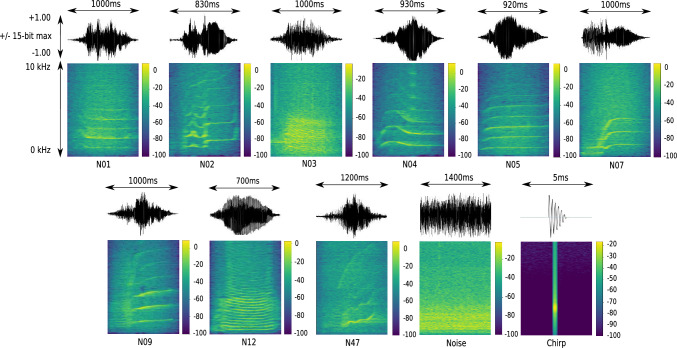
Killer whale call types, interfering/added boat noise, and the chirp signal, all of them utilized in the experimental setup of ORCA-SPY.
3. (EXP-3) aims to simulate a variety of realistic scenarios for a large combination of varying distances (200 m to 1,000 m), water depths (0 m, 100 m, 200 m), and added interfering boat noises, at $\Delta$3$^\circ$ intervals. These combinations lead to an SNR-scale of $+$3 dB to −14.2 dB.
4. (EXP-4) simulates the effects of drifting and sinking streamers. During previous fieldwork expeditions^46^ measurements were always performed during moderate driving speed, since driving too slowly caused a sink and/or drift of the hydrophone streamers. Drifting can occur during a change of course or in a strong current at a low speed. However, sinking depends on the speed of the moving boat as well as the buoyancy of the hydrophone array. Both streamers typically sank by about 3.5$^\circ$ at a moderate speed of 2.5 knots according to a depth sensor attached to the streamers. In general, killer whale localization is disabled in case the travel speed is too slow. Nevertheless, experimental simulations are mandatory to estimate the impact of drifting and/or sinking array streamers in terms of localization accuracy.
5. Whereas (EXP-1) through (EXP-4) primarily focus on the detection and localization of single isolated killer whale vocalization events, (EXP-5) addresses multiple vocalizing individuals with and without an added interfering noise signal originating from a boat with an SNR range of $+3$ to $-3$ (see Supplementary Figure S2).

**Figure 4:**
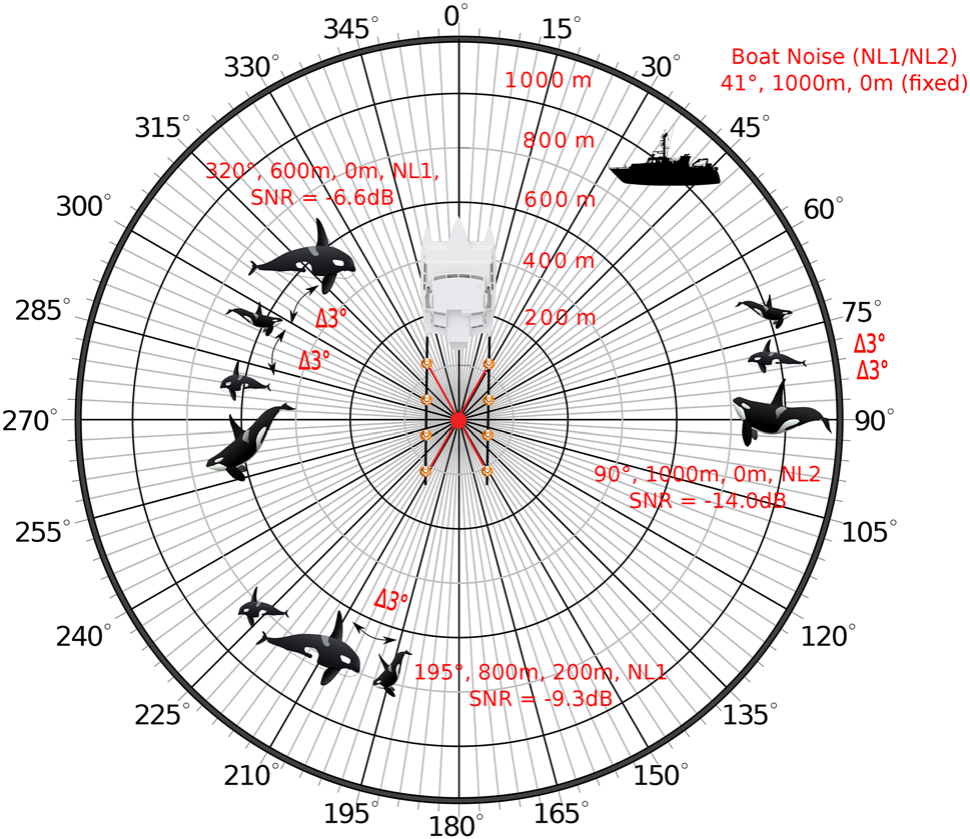
Position of orca (SL = 156 dB re. 1 $\mu$Pa p-p) and interfering boat noise (NL1 = 167 dB re. 1 $\mu$Pa p-p or NL2 = 170 dB re. 1 $\mu$Pa p-p, constant position of 41$^\circ$). Depicted are the three followings examples. (1) An orca at $90^\circ$, 1000 m distance, and 0 m depth with a noise interference of NL2 would result in an SNR of −14.0, (2) An orca at $195^\circ$, 800 m distance, and 200 m depth with a noise interference of NL1 would result in an SNR of −9.3, and (3) An orca at $320^\circ$, 600 m distance, and 0 m depth with a noise interference of NL1 would result in an SNR of −6.6, see also Supplementary Figure S1 and Supplementary Table S6.

A set of scenario examples are visualized in Fig. 4 with their respective combinations of distance, depth, source, and noise levels. Table 1 shows a list of all simulated experiments ((EXP-1) to (EXP-5)) performed within the scope of this study regarding complexity concerning varying combinatorial assemblies.

**Table 1:** List of all different experiments (EXP-1 to EXP-5) performed within the simulations of this study.

Experiment	Signal Types	Distance (m)	Direction ($^\circ$)	Depth (m)	Array setup	Noise interference and SNR-scale	Purpose
EXP-1	2 ms Dirac- like chirp	200	0, 3, 6, ..., 357	0	Short, Long	No added noise	Excluded systematic error, best possible result, directional dependency
EXP-2	N01–N05, N07, N09, N12, N47	200 400	0, 3, 6, ..., 357	0, 100, 200	Short, Long	No added noise, added no1 noise with SNRs ranging from $+$3 dB to −4 dB	Accuracy regarding different call type structures
EXP-3	N01 N04 N09	200 400 600 800 1000	0, 3, 6, ..., 357	0, 100, 200	Short, Long	No added noise, added no1, no2 noise with SNRs ranging from $+$3 dB to −14.2 dB	Accuracy regarding different SNR ranges
EXP-4	N01 N04 N09	200 400 600	0, 3, 6, ..., 357	0, 100, 200	Long, $a = 15^\circ ,$ $e = -3^\circ ,$ $-5^\circ , -90^\circ$	No added noise, added no1 noise with SNRs ranging from $+$3 dB to −7 dB	Accuracy robustness regarding array drifting errors
EXP-5	N01 N09 N47	200 400	40, 125, 220	0	Long	No added noise, added wh1 with SNRs ranging from $+$3 dB to −3 dB (interfering boat at bearing 305$^\circ$)	Accuracy regarding overlapping calls

The experiments differ in complexity with respect to varying combinatorial assemblies regarding: (1) signal types—chirp sound versus diverse numbers and types of noisy orca calls (see Fig. 3), (2) distance source origin—combinations of various ranges from 200 m to 1000 m, (3) direction source origin—120 equidistant positions ($\Delta$3$^\circ$ per emitted signal), (4) water depth source origin – mix of different ranges from 0 m, 100 m, up to 200 m, (5) hydrophone array setup and positioning – short versus long, together with varying azimuth *a* and elevation *e* (see Fig. 1), and (6) noise interference through four interferences. Either no noise interference, a static light white-noise interference (wh1), a small ship with medium noise interference (no1), a ship with strong noise interference (no2) added to the original noisy orca call (see Fig. 3) leading to various SNR-scales, at a total range of $+$3 dB to −14.2 dB.

### Summary of field deployment scenarios

The lake Stechlin experiment was performed in preparation for the DeepAL 2022 expedition. Both the lake Stechlin experiment and the DeepAL expedition were supposed to assess the ORCA-SPY tool chain in live conditions and compare the results of the simulated environment with real-life environments.

#### Lake Stechlin

The Lake Stechlin experiments (EXP-6) tested ORCA-SPY on the Lake Stechlin Array, as depicted in Fig. 2, under laboratory conditions. As there was no ship with a generator available, the Stechlin array was deployed as a fixed array via a footbridge as depicted in Fig. 6 instead of being towed. During the recording sessions, a small electric motorboat was driven clockwise around the array. Every 30$^\circ$ the boat was anchored, and a localization test file containing different Orca calls was played for 3–5 min through an underwater loudspeaker at 60 dB strength. The GPS positions of the three recording sessions are depicted in Fig. 6. The replay of Orca calls was considered to be harmless to the existing animals, and permission for the experiment was given by the Leibniz Institute of Freshwater Ecology and Inland Fisheries (IGB)^49^.

#### Lake Stechlin challenges

1. The second hydrophone from the top of the left streamer of the Lake Stechlin array in Fig. 2 was found to be faulty after the deployment. A strong electronic interference in the lower frequency range (below 1.5 kHz, as depicted in the example signal in Fig. 5a. Figure 5b displays the same time frame from another hydrophone for reference) was found to interfere with the localization results. Due to time constraints, the damaged hydrophone could not be replaced on-site. A 1.5 kHz high-pass filter was applied during offline evaluation to remove the electronic interference to reduce the effect on the localization.

**Figure 5:**
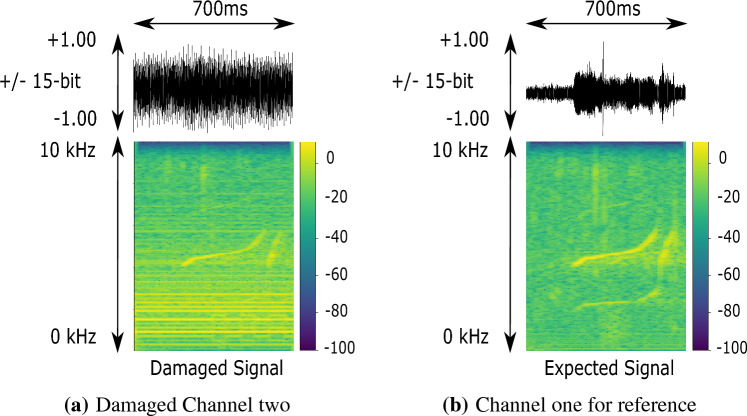
Raw Data depictions of the strong electronic interference during the lake Stechlin expedition on channel two on the left. The same time frame of channel one is depicted on the right for reference.
2. Shipping traffic did not stop on lake Stechlin. Thus, distant engine noises were unavoidable during the recording sessions. In addition, multiple boats were docked to the footbridge at all times and a loud interference noise was created if a docked ship was hit by a wave or collided with the footbridge. To combat the effect of the above mentioned interfering noises, a band-pass filter between 500 Hz and 10 kHz was applied on all channels. None of the noise interferences which were encountered was similar enough to the orca target signal to create false positive detections.
3. The Streamers were aligned as parallel as possible under the circumstance, but due to constraints during deployment, the height between the streamers were found to be slightly different on-site. These differences, as depicted in Fig. 2, were taken into account for the calculation of the origin of the reference coordinate system.
4. Due to the restricting cable length and power supply, we could not deploy the testing network far from the shore and as such were only testing in shallow waters with a depth of less than meters.
5. To construct said reference coordinate system, the GPS positions of the beginning and end of both streamers were taken. But due to GPS inaccuracies, the alignment of the hydrophone array was ambiguous in comparison to the signal position. As such, we did know the GPS position of the signals and the array, but not the ground truth bearing of the signal in comparison to the fixed array 0$^\circ$ (12 o’clock, Front) direction during the recording. This inaccuracy was corrected by assuming that the measurements’ error adhere to a Gaussian distribution. Under this assumption, the sum of the signed bearing error directions of the measurements should be zero. Using this assumption, the most likely hydrophone array alignment can be estimated by calculating the signed bearing error directions of a realistically chosen arbitrary array alignment and by iteratively adding the mean signed bearing error direction to the arbitrary array alignment to get an improved array alignment until the sum of signed bearing error directions is approximately zero (Fig. 6).

**Figure 6:**
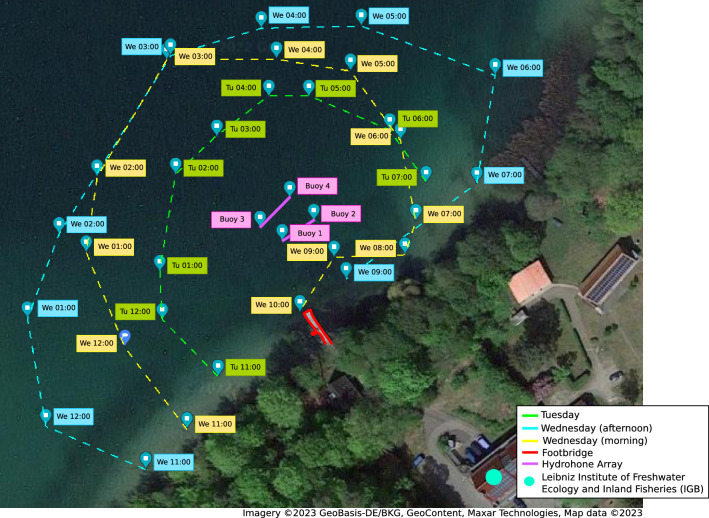
A depiction of the GPS positions from the lake Stechlin recording sessions. The three sessions were conducted over a time span of two days. The first sessions was on a Tuesday and is depicted in green. The second and third sessions were conducted on the following Wednesday morning and afternoon. The morning session is depicted in cyan, the afternoon session is depicted in yellow. The array was positioned between the bouys in the center of the Figure. The two streamers are depicted in orange. The array was connected via the footbridge outlined in red.

#### DeepAL 2022

The DeepAL 2022 expedition deployed ORCA-SPY on the DeepAL2022 array depicted in Fig. 2. The array was powered and towed by the expeditionary research vessel Tomorrow. While the deployment of ORCA-SPY in the field was of high interest, the main focus of the expedition was to gather new material for further scientific studies.

#### DeepAL 2022 expedition challenges

A ground truth bearing of a target animal is required in order to evaluate ORCA-SPY localization accuracy. The problem is that in our passive acoustic monitoring approach, the encountered animals do not have any tracking device which could be used to get a comparable ground truth. We tried to use visual confirmation to infer the localization accuracy, but our method was too imprecise to be used in numerical calculations. In addition, a similar experiment such as the Lake Stechlin setup was ethically unreasonable, since the noise pollution of an 60 dB underwater loudspeaker could be harmful to the existing animals. During the expedition we encountered fog bells, buoys with a loud audible bell and a number designation which can be used to find one’s position on a map in case of heavy fog. Interestingly, ANIMAL-SPOT was never trained on bell sounds and the fog bell signal with its higher harmonics, as displayed in Fig. 9, was in proximity similar enough to our orca target signal, so that ANIMAL-SPOT could be tricked into false positives with a lowered threshold of 0.40. This revelation gave us the idea to evaluate the ORCA-SPY localization accuracy using fog bell buoy 33 as a reference, since the GPS position of the fog bell was known. But the approach also had some downsides:

1. The fog bell buoys are positioned along shipping routes, making it hard to find a window to evaluate without interference.
2. The bell signal is weak in comparison to an animal call, so ANIMAL-SPOT can only falsely detect the signal in close proximity and without noise interference.

**Figure 7:**
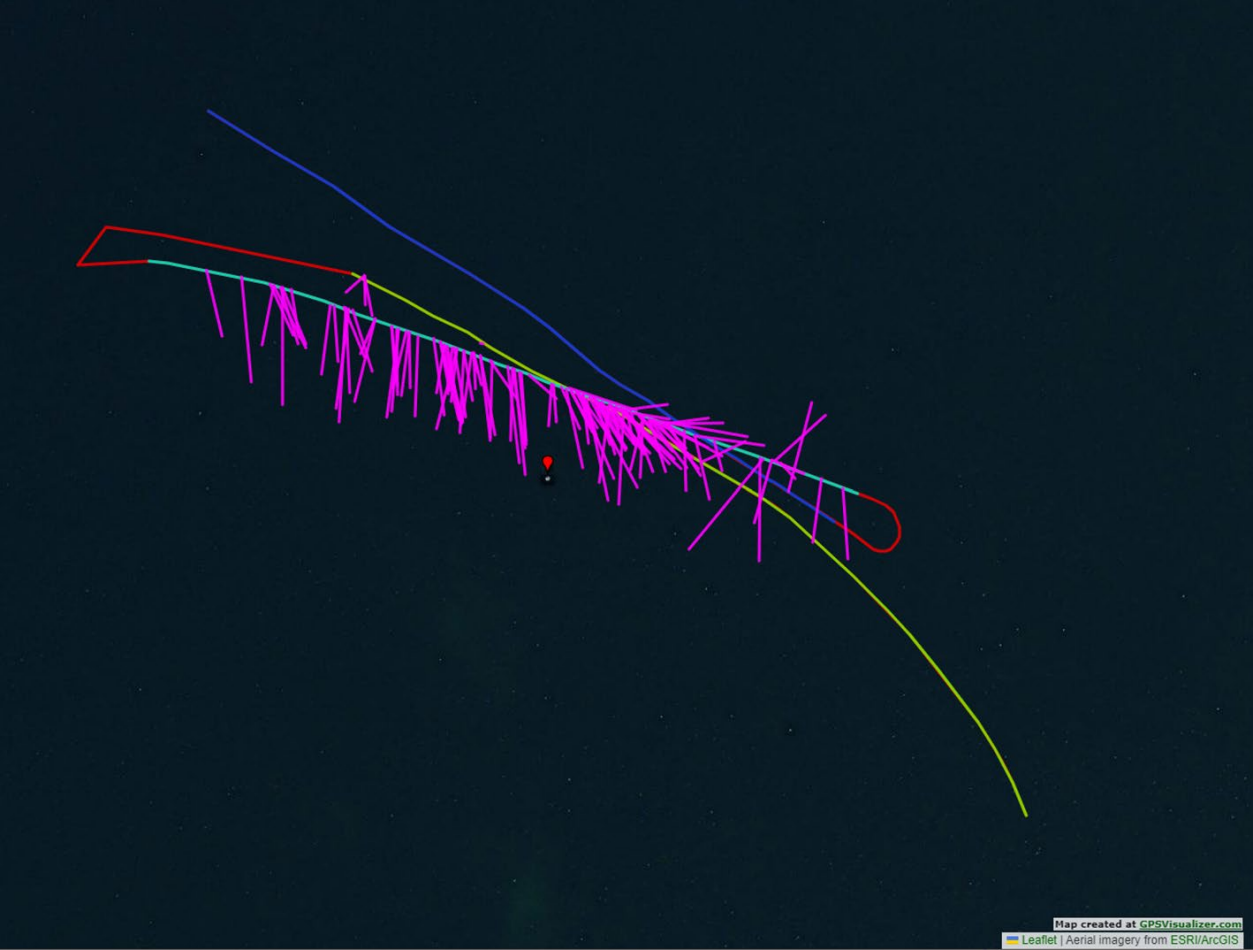
A depiction of the GPS route, recordings, and localizations from the first encounter with the fog bell buoy 33 . The encounter was split into three recording sessions, which were conducted in separate passes. During the first pass in light green there was a motorboat interference making it impossible for ANIMAL-SPOT to detect the fog bell buoy. The motorboat left during the second pass in cyan, which yielded the most localization results depicted in pink for the first encounter. During the later half of the second pass, a new interference ship was passing by in the north-east (right to top). The effect of the interference can be seen during the end of the second pass and the beginning of the third pass in blue. Much like the first pass, the third pass had too much interference, making it impossible for ANIMAL-SPOT to detect the fog bell buoy signal.

**Figure 8:**
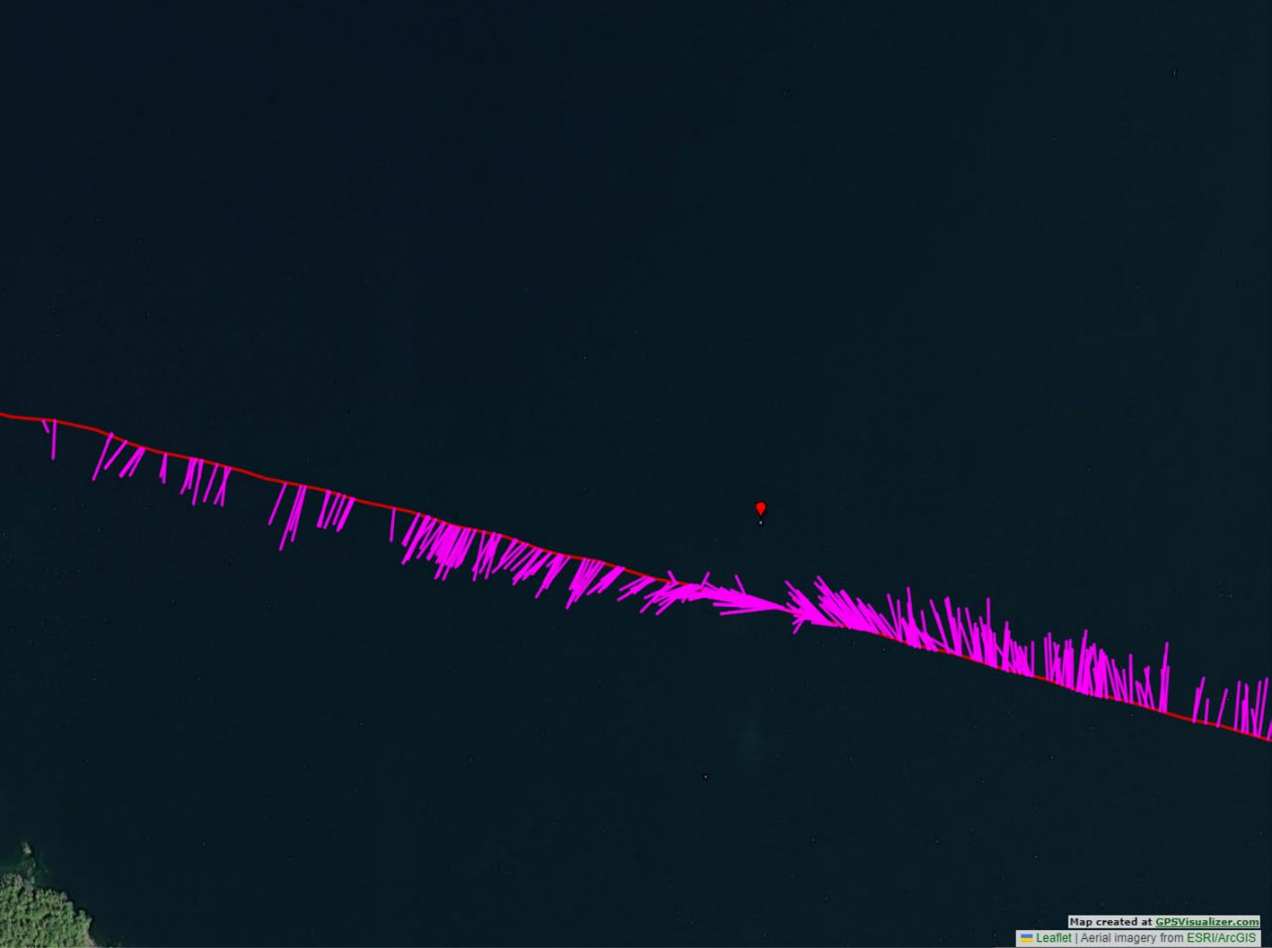
A depiction of the GPS route and localization results from the second encounter with the fog bell buoy 33 going from the right to the left. This encounter was only a drive pass from a longer distance. After we passed the fog bell, a motorboat was moving around the island in the bottom left corner. Interestingly, while the motorboat noise was interfering with our localization result, ANIMAL-SPOT was able to detect the fog bell signal despite the interference. This phenomenon can be interpreted in a way that the fog bell signal was still detectable for ANIMAL-SPOT in the power spectrogram, but no longer the most energy significant part for the TDOA localizer to localize.

As such, two recording sessions with fog bell buoy 33 were deemed enough for a prove-of-concept. Figures 7 and 8 display the GPS positions and localization of the two recording session.

## Results

**Figure 9:**
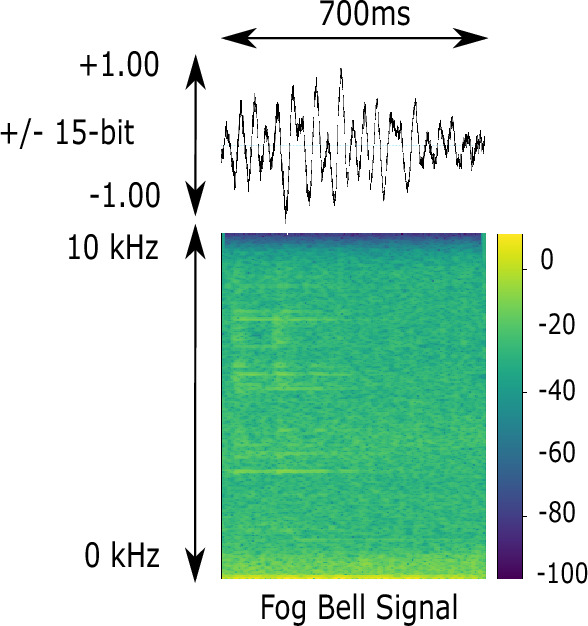
A depiction of the audio spectrum of the underwater fog bell buoy 33 signal during the DeepAL22 expedition. The signal is weak in comparison to possible noise sources such as motorboats. As such, localization was only possible in close-proximity and without interference in the area.

An inter-comparison with other research studies is difficult due to: (1) different data repositories, including varying data processing and preparation techniques (e.g., other species, type and amount of data, ground truth, etc.) and (2) various non-comparable approaches (e.g., other concepts/use-cases, array setups, localization scenarios, evaluation metrics, software frameworks, etc.). In addition, ORCA-SPY’s accuracy is dependent on the underlying modules used during evaluation. As such, the TDOA bearing calculation results depend on the ANIMAL-SPOT detector and bearing calculator implementations, which can be replaced in future developments (Fig. 9).

### Simulated results

**Figure 10:**
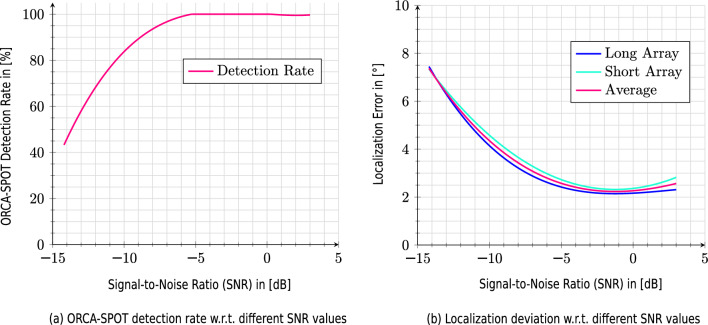
Detection rate of ANIMAL-SPOT (**a**) and localization error (**b**), both depending on the SNR value. All curves were generated via 3rd-degree polynomials (spline interpolation) with respect to the point-wise SNR-based results achieved in EXP-3, considering all combinatorial options, leading to an entire SNR-range of $+$3 dB to −14.2 dB. The localization error slightly increases regarding the best SNR-values, because in this case ANIMAL-SPOT identifies even frames containing very small portions of call activity as valid killer whale segments, which in turn leads to more frame-specific localization outliers and consequently higher average errors.

A comprehensive analysis of the results of every experiment can be found in Supplementary section Simulated Experiments. To summarize, across all simulated experiments utilizing 58,320 embedded vocalizing killer whale events, subjected to various hydrophone array geometries, call types, distances, and noise conditions responsible for a signal-to-noise ratio varying from -14.2dB to 3dB, ORCA-SPY achieved a detection rate of 94.0 $\%$ with an average localization error of 7.01$^\circ$. The ANIMAL-SPOT detection rate and localization error are both dependent on the signal-to-noise (SNR) ratio and can be described as a function, as depicted in Fig. 10 from the results of (EXP-3).

### Field experiment results

The challenges and numerical uncertainties discovered during the live deployment of the Lake Stechlin experiment (EXP-6) and the fog-bell passes during the DeepAL 2022 expedition (EXP-7) also express themselves in the results. In the case of the lake Stechlin deployment of (EXP-6) 3889 detection events were processed from the 31 recording sessions with 3 hours of data material performed in Fig. 6 with a mean accuracy error of 29.19$^\circ$ and a median of 17.54$^\circ$. Nonetheless, the knowledge acquired during (EXP-6) was useful to improve the handling and deployment of the array by applying new strategies such as the band-pass filter, which in turn improved the accuracy and authenticity of the ORCA-SPY performance in the DeepAL22 expedition. During the two recording sessions of 1.1 hour length of fog bell buoy 33 , depicted in Figs. 7 and 8, 503 detection events were processed with a mean accuracy error of 20.01$^\circ$ and a median of 11.01$^\circ$.

## Discussion

In real-world recordings, there is an increasing problem of potential false alarms due to different animal vocalizations and a generally larger signal variety, altogether causing localization outliers. Consequently, it is particularly important to address the detection limitations in real-life scenarios while running ORCA-SPY as an application on research vessels. Generally, ANIMAL-SPOT was verified in large-scale evaluation scenarios, while achieving a false-positive rate of $\approx$4 %, together with a precision of $\approx$93 %^46^. Deep learning-based binary-class segmentation reduces the number of false alarms and consequently the number of errors in the localization. Nonetheless, some false alarms may still occur, resulting in high localization errors. In general, ORCA-SPY is a conceptual study to analyze and evaluate the approach of combining deep-learning-based killer whale signal identification (see ANIMAL-SPOT^46^) with simultaneous TDOA-based sound source localization, all together embedded within PAMGuard’s^45^ software framework as a fully automated workflow. However, the underlying modules used within this approach are interchangeable and adaptable to other active/passive recording constellations and target signals. In this study, the simulated scenarios were verified and designed with a towed hydrophone array in mind. The scenarios were created in accordance with recording setups of previous fieldwork expedition (DeepAL19)^46^ and preparations for follow-up expedition (DeepAL22) (Table 2).

**Table 2:** A summary of all the experiments, EXP-1 through EXP-5 were simulated using ORCA-SPY and SimSAcq. The Detection rate is the ANIMAL-SPOT detection rate, the Localization accuracy is the absolute mean Bearing Calculaiton error.

Experiment	Description	Detection rate	Localization error
EXP-1	Dirac-pulse-like signal	100.0%	0.04$^\circ$
EXP-2	Call type structures	98.2%	7.01$^\circ$
EXP-3	High-SNR-range	90.6%	3.81$^\circ$
EXP-4	Changing array (sinking)	98.2%	7.16$^\circ$ (89.86$^\circ$)
EXP-5	Overlapping calls	100.0%	7.76$^\circ$
EXP-6	Lake Stechlin	100.0%	17.54$^\circ$
EXP-7	DeepAL 2022	n.a.	11.01$^\circ$

The results of (EXP-1) in Supplementary section Simulated Experiments indicate that the long array yields better results than the short array. This was expected, due to the larger distances between hydrophones (see Supplementary Figure S3). In addition, Supplementary Figure S3 visualizes that the localization error depends on the angle and hydrophone array composition. In the case of 0, 90, 180, and 270 degrees the localization errors are smaller, whereas, in comparison to 45, 135, 225, and 315 degrees the angles depict larger deviations with respect to the ground truth. Under noisy conditions, similar tendencies can be observed. The prediction is more accurate if the vocalizing event arrives either directly from the left, right, front, or back of the hydrophone array. The tendencies are displayed in Supplementary Figure S3c–h when considering the direction of the research trimaran (see Fig. 1). The constant location (41$^\circ$) of the interfering ship noise (see Fig. 4) has no impact on the symmetric properties of the localization errors, which has been proven by the results of the noise-free chirp signal in (EXP-1) (see Supplementary Figure S3a and b). Consequently, these symmetrical properties of localization errors are caused by the actual hydrophone geometry. In all 4 cases—0$^\circ$ (front), 90$^\circ$ (right), 180$^\circ$ (back), and 270$^\circ$ (left)—the signal arrives in a first approximation simultaneously on more than one hydrophone. In the case of 90$^\circ$ (right) and 270$^\circ$ (left), the signal hits 4 hydrophones at the same time, whereas for 0$^\circ$ (front) and 180$^\circ$ (back) 2 hydrophones are affected simultaneously. Such situations facilitate an approximation of corresponding positions and therefore result in better localization accuracies. However, all other directions lead to distinct appearance times per hydrophone, whereas the diagonals maximize those different arrival times per hydrophone and consequently show the largest localization errors/outliers. (EXP-1) provides not just the best possible error and consequently an ideal lower accuracy boundary (short hydrophone array of 0.205$^\circ$, long hydrophone array of 0.035$^\circ$) which can be achieved through ORCA-SPY, but also proves no directional tendencies with respect to the localization algorithm (see symmetric properties in Supplementary Figure S3a,b).

The SNR values occurring in (EXP-2) (see Table 1, SNR $+$3 dB to −4 dB) do not present a problem in terms of detection robustness, which is also shown and illustrated in Fig. 10. ANIMAL-SPOT’s detection accuracy starts declining slowly at about −6 dB to −7 dB. However, the varying call type structures have a stronger impact on the final localization accuracy (see Supplementary Table S3). Killer whale call types N05 and N12 have an unusually high error compared to the other vocalization patterns, which can be explained through their call type structure (see Fig. 3). N05 and N12 have a very constant appearance, which makes the results regarding TDOA less reliable, especially if the segmentation window starts in the middle of the call. The remaining killer whale vocalization types (see Supplementary Table S3) are of comparable magnitude in terms of the overall localization error. If the N05 and N12 call patterns were excluded from the calculation of the overall call type specific localization error/deviation (see Supplementary Table S3), a final average error of 4.31$^\circ$, compared to 7.01$^\circ$, would be achieved with respect to the remaining 7 call types. The removal of the two best and two worst call types lead to an overall call type specific localization deviation of 5.15$^\circ$ on average. Besides the information in Supplementary Table S3, Supplementary Figure S3c–f visualizes the best (N47), medium (N03), worst (N05), and the average call type related, and hydrophone array (short/long) specific, localization errors, evaluated across all parametric constellations of (EXP-2).

Besides the different call type patterns, the detection frame and the consideration of multiple findings (see Supplementary Table S2) impact localization. Supplementary Table S3 illustrates that the last detection frame of a killer whale vocalization event has worse results than the first and second detection concerning triple findings. This is also reflected in the double findings, where the first detection frame is also consistently better than the second. A reasonable explanation for such a trend is: (1) the first detection frame usually starts with a small noise offset followed by the actual killer whale vocalization, whereas the last detection frame normally begins within the call, and (2) all frames between the first and the last (only in case of triple and/or more than three findings) generally contain larger amounts of killer whale signals. Consequently, the first and all in-between windows perform better (as shown in Supplementary Table S3), compared to the last detection frame, which in turn reduces the TDOA-based localization accuracy significantly. The results of (EXP-3) also substantiate previous observations concerning (EXP-1), showing that the long array leads to smaller localization errors compared to the short array (see Supplementary Table S7 and Supplementary Figure S3 (g)  & (h)), across all possible parametric constellations, covering an entire SNR-scale ranging from $+$3 dB to −14.2 dB (see Supplementary Equation 1 and Fig. 10), with respect to the most frequent occurring call types, according to Ness^9, 47^. Supplementary Figure S1 visualizes example spectrograms of an N09 call type under various SNR conditions, whereas Supplementary Figure S1b is an example of clipping. Due to a relatively high gain (see Supplementary Table S1), especially for killer whales that are not far away (small attenuation), together with a strong noise source level, intentional clipping is caused at times, although this does not have a major impact on the localization accuracy. This is a very important finding because previous fieldwork experience has shown that clipping may occur due to a variety of unforeseen reasons.

Moreover, Fig. 10 clearly proves ORCA-SPY’s robustness regarding killer whale signal segmentation as well as localization, still within very noisy conditions. Even at $\approx -$9 dB, about 90 % of all killer whale vocal activities are detected, which results in an average localization error of about $\approx$3.8 degrees, considering ANIMAL-SPOT’s pre-defined confidence threshold of $>=$ 0.92. Adjusting the threshold allows sufficient killer whale events to be detected at even worse SNR values than displayed in Fig. 10a, although the trade-off between precision and true-positive rate must be taken into account here. An interesting observation can be made in Fig. 10b (see also Supplementary Table S7), where the mean localization error becomes slightly smaller even with respect to progressively worse SNR, ranging from $+$3 dB to $\approx -$2 dB. In addition, larger localization errors occur within increasingly worse SNR scenarios (steep gradient), despite the fact that ANIMAL-SPOT still identifies enough events required for robust localization. These two phenomena can be addressed and justified via two hypotheses: (1) high and/or slightly poor SNR ratios (see $+$3 dB to $\approx -$2 dB) result in large numbers of detection frames per vocalization event, because even weak orca signals are enough to encounter killer whale frames, which strongly increase the probability concerning the previously mentioned trend regarding multiple findings and accompanying growing localization outliers, and (2) significantly worse SNR situations (see $\approx \,<-$9.5 dB) make it gradually harder for the localization algorithm to decide whether to focus on the orca signal or interfering noise source, thus leading to severe rises of the actual localization error. Whereas hypothesis (1) has been already proven (see results EXP-2), the $2^{nd}$ statement was analyzed and verified by running additional experiments on 1200 m combined with noise interference. In those cases, the localizer reliably detects the interfering boat at 41 $^\circ$ (see Fig. 4).

Since this does not give us any valuable information on the accuracy of our tool chain, we decided to only estimate the direction up to 1000 m.

The results of (EXP-4) indicate that minor changes (both, drifting and sinking events) have only a small influence on the localization performance. Supplementary Table S4 illustrates that the loss concerning accuracy compared to the exact position for the streamers sunken by $-3^\circ$ is only about 0.1$^\circ$ – 0.2$^\circ$, 0.5$^\circ$ – 0.6$^\circ$ with respect to −5$^\circ$ sinking, and 4$^\circ$ – 5$^\circ$ regarding 15$^\circ$ drifting. In the most extreme situation (no movement, the towed streamers sink down to the bottom), the localizer is still capable of distinguishing between the starboard and port side. However, if the sound source is above the hydrophone (0 m depth), the source is estimated to be in front of the ship. If it is below (100 m and 200 m depth), the source is estimated to be behind the ship, which explains the large errors/deviations regarding the 90$^\circ$ constellations (see Supplementary Table S4).

As already discovered through (EXP-2) and (EXP-3), the bearing calculation is dependent on the most prominent (highest RMS intensity) feature within an ANIMAL-SPOT segment. Within the scope of (EXP-2) through (EXP-4) the prominent feature was SNR and call type structure driven. The (EXP-5) scenario was designed with multiple targets in one ANIMAL-SPOT segment in mind. The results of (EXP-5), displayed in Supplementary Figure S2, have shown that ORCA-SPY can localize the most prominent call in a window with multiple calls present. With a mean accuracy error of 7.76$^\circ$, this current ORCA-SPY approach suffices for the DeepAL expeditions as it has no need to differentiate between call source locations while in the field. The field deployments during (EXP-6) and (EXP-7) have proven that ORCA-SPY can be used to find, track and follow a target signal even in noisy environments. Yet, the expeditions have also shown that there are large differences between the simulated data of the SimSAcq module and real data. Most of the differences were expected, such as the recording quality between different hydrophones and the dynamic range. The simulation was based on mono-file recordings, thereby depicting an ideal hydrophone array of hardware identical hydrophones, which does not exist in reality. Yet those expected differences could not have a strong negative influence on the localization accuracy, since the dynamic range of a hydrophone only affects the strength of an incoming signal, but not its characteristics.

This is also confirmed with regard to the detection samples of (EXP-6) and (EXP-7). From the 3889 of (EXP-6) and 503 localizations of (EXP-7) 1461 (1148 from (EXP-6) and 313 from (EXP-7)) samples had an accuracy error of less than 15$^\circ$ regarding the ground truth. This indicates that 33.26$\%$ of the recorded data of (EXP-6) and (EXP-7) are comparable to the simulated examples of (EXP-2) and (EXP-3). As such, the problematic differences between the simulated data are not necessarily systematical in nature with the approach of ORCA-SPY, but situational. As already stated during (EXP-5), the built-in bearing localizer only calculates the bearing of the most intensity significant feature in an ANIMAL-SPOT segment window. Dirac-impulse like interferences, such as motor cavities, mechanical hits, or electrical dropouts, with a higher SNR value than the target signal, are falsely preferred in the localization. Those interferences were not taken into account during the simulation as they depend heavily on the used hardware, power supply, and the surrounding area. In order to counteract the effects of said interferences, the FFT spectrogram engine noise removal tools, as well as the band-pass filter between 500 Hz and 10 kHz were applied, yet with regard to this study about 2931 real data samples (or 66.74$\%$ of real data recordings) were affected by Dirac-impulse like interferences or interferences with a higher SNR than the target.

## Conclusion and future work

In this study, a deep-learning-based sound segmentation module, named ANIMAL-SPOT^46^, was embedded as a PAMGuard module in order to build ORCA-SPY. ORCA-SPY is a sound source localization and simulation framework for real-world killer whale identification, and was evaluated within a large-scale experimental built-up in order to simulate real-world scenarios. The framework was successfully tested and deployed in two expeditions. ORCA-SPY found 54,798 out of 58,320 vocalizing killer whale events concerning (EXP-2) and (EXP-3), across various orca call type patterns within SNR scenarios ranging from $+$3 dB to −14.2 dB, thus achieving an average detection rate of 94.0 %. The average localization error across all 9 different call types was 7.01$^\circ$ (see results EXP-2). Considering only the three most frequent calls along all possible combinatorial variations (see results EXP-3) yielded an average localization deviation of 3.81$^\circ$.

The first field deployment (EXP-6) of ORCA-SPY was performed at Lake Stechlin in laboratory conditions and yielded an average error of 29.19$^\circ$. The error was significantly higher than what would have been expected from the simulations, since this test was primarily designed to find possible problems in a natural setting to prepare for the DeepAL22 expedition (EXP-7). During the said expedition, the average localization error was improved to 20.01$^\circ$ using the know-how acquired during the Lake Stechlin experiment.

Further work has to be done to reduce or to remove strong SNR and Dirac-impulse like interferences or develop a localizer with higher robustness against said interferences. There already exist two projects to solve the annotated problems, (1) ORCA-CLEAN^59^ a deep neural network designed to denoise audio data based on a target signal and (2) ORCA-SCAN^60^ a deep neural network localizer designed to take both DOA and phase information into account to improve localization. Both modules have, to the best of the author’s knowledge, not yet been deployed in a live experiment or integrated into a framework such as PAMGuard^45^. Moreover, ANIMAL-SPOT^46^ has been modified in a way that it is capable of handling any other bioacoustic signals, either within binary target/noise segmentation and/or multi-class species/call type identification scenarios. ANIMAL-SPOT^61^ was evaluated on 10 different species as well as 1 genus and the raw deep learning module was already integrated and is available in PAMGuard^45^ and consequently also within the ORCA-SPY framework. Thus, it is possible to use animal-dependent detectors/classifiers fine-tuned to any other vocalizing species, combined with the entire functional repertoire available in PAMGuard^45^. The raw deep learning module also functions as a basis for further development, in order to seamlessly integrate newly trained ANIMAL-SPOT versions based on advanced network architectures such as ResNeXt^62^ and HrNet^63^. Another approach that is currently under development is the integration of other viable detection or localization methods such as Memristor based sound localization^64^ or FIN-PRINT^65^, a fully automated framework for the individual recognition of killer whales in pictures. Such an addition could introduce new multi-modal possibilities for improvements in detection, localisation and classification. This is similar to the DMMAN network described by Hu et al^66^, which would not only improve the performance of ORCA-SPY, but would also help with target differentiation for context dependent analysis with towed and stationary observation. ORCA-SPY generalizes in a way that it allows researchers to simulate and verify various array geometries and setups under assumed realistic real-world noise conditions, which is not just important in the field, but also in preparation for any fieldwork studies. Applying ORCA-SPY in the field, it is possible to assign and map animal-related sound events to the corresponding animals, assuming the single individuals are sufficiently far apart from each other and the SNR values of interferences are below the SNR of the target signal. In this way, individual-specific data repositories can be created to analyze dialects, speaker identification algorithms, and turn-taking events to gain deeper insights into the world of animal communication.

The entire ORCA-SPY framework will be part of upcoming fieldwork studies. Deep learning based killer whale detection utilizing ANIMAL-SPOT^46^ has already been successfully implemented and tested in the 2019 fieldwork expedition, clearly demonstrating that network generalization and accuracy, in addition to real-time processing, is not only possible but also extremely promising and helpful. ORCA-SPY and all PAMGuard related code extensions, the ANIMAL-SPOT source code, will be publicly available here^48^.

## Supplementary Information

Supplementary Information.

## Data Availability

All PAMGuard related code extensions, the ANIMAL-SPOT source code, along with the chosen killer whale vocalization patterns utilized in this study, are documented and available here^48^. Moreover, the repository^48^ provides a detailed user guide to generate an animal-specific simulated data corpus, besides a proper setup of the entire ORCA-SPY framework and exemplary test scenario. The Orchive^9, 51^ as well as the Call Type Data Corpus (CTDC)^67–69^, both collected by the OrcaLab^51^ and Stephen Ness^9, 50^, is publicly available, only in agreement with the OrcaLab^51^ and Steven Ness^9^.
