# Supplementary material for: ORCA-SPY enables killer whale sound source simulation, detection, classification and localization using an integrated deep learning-based segmentation
Source: Sci Rep. 2023 Jul 10;13:11106. doi: 10.1038/s41598-023-38132-7 (PMC10333356; doi:10.1038/s41598-023-38132-7)
Supplement: Supplementary file 1 — Supplementary Information. [file 41598_2023_38132_MOESM1_ESM.pdf]

ORCA-SPY enables killer whale sound source simulation, detection, classification and localization using an integrated deep learning-based segmentation

Christopher Hauer[1,*], Elmar Nöth[1], Alexander Barnhill[1], Andreas Maier[1], Julius Guthunz[6], Heribert Hofer[2,3,4], Rachael Xi Cheng[2], Volker Barth[5], and Christian Bergler[1,*]

[1]Pattern Recognition Lab, Department of Computer Science, Friedrich-Alexander-Universität Erlangen-Nürnberg, Martensstr. 3, 91058 Erlangen, Germany
[2]Leibniz Institute for Zoo and Wildlife Research (IZW), Alfred-Kowalke-Straße 17, 10315 Berlin, Germany
[3]Department of Veterinary Medicine, Freie Universität Berlin, 14195 Berlin, Germany
[4]Department of Biology, Chemistry, Pharmacy, Freie Universität Berlin, 14195 Berlin, Germany
[5]Anthro-Media, Nansenstr. 19, 12047 Berlin, Germany
[6]Universität des Saarlandes, 66123 Saarbrücken, Germany
[*]christian.bergler@fau.de, Hauechri.Hauer@fau.de

Supplementary Information

Simulated Experiments

The following attachment describes the experimental framework in order to analyze and evaluate ORCA-SPY's localization accuracy and robustness. A large-scale data collection of the different killer whale-, array-, and position-specific ground truth localization data presents significant challenges and is not readily feasible. Therefore, the simulated experiments are exclusively based on simulated localization data in the form of realistic multichannel underwater audio streams. The simulated scenarios follow a variety of real-world situations encountered during previous fieldwork expeditions[1] embedding various killer whale sound events, produced by ORCA-SPY's sound source simulation framework. For the simulated experiments, all killer whale call types were embedded as examples (see Figure 3) into multichannel underwater audio streams. The calls are per se stand-alone calls without any other overlapping patterns. However, (EXP-5) also cover scenarios with multiple vocalizing killer whale individuals, ending up in very short consecutive and/or overlapping sound events.

Hydrophone Array Composition

Within the scope of the simulation, two different hydrophone array geometries were utilized, which were inspired by the recording setups of previously conducted fieldwork expeditions[1]. Thus, it was ensured that the simulated localization scenarios are as close to reality as possible, which in turn guaranteed reliable preparation with respect to future fieldwork expeditions. These two planar hydrophone geometries were verified under various parametric constellations, together with all relevant software components, in order to analyze their accuracy. Figure 2 visualizes the long and short towed-hydrophone geometries used during the simulation. In future fieldwork expeditions, the hydrophone streamers would be attached to the two outrigger hulls of a research vessel. The streamers were separated by 7.236 m (23.74 feet) and four hydrophones were embedded in each streamer. Figure 2 shows the distance to the boat hull regarding hydrophones H0 and H4, the (x, y, z)-coordinates for each hydrophone with respect to the center of the array are depicted in Supplementary Figure S2. Except for setup EXP-4 (see below), the array is assumed to float on the surface of the water and be aligned with the direction of the vessel. Consequently, this results in an azimuth a (Figure 1 - top view), elevation e (Figure 1 - side view), and depth z (Figure 1 - top view), which are all equal to zero. The hydrophones of the short array (S) are approximately 1.5 m apart, whereas the long array (L) shows a distance of roughly 2.8 m. The first hydrophones (H0, H4) are 29.3 m behind the end of the trimaran. Hydrophone H0 was utilized as the input channel in order to distinguish between killer whale vocalizations versus environmental noise, performed by ANIMAL-SPOT[1].

Target Signals and Background Noise

To demonstrate and test the robustness of the multi-module-based, sequentially, and chronologically ordered sound source localization framework ORCA-SPY, including (1) sound acquisition, (2) data pre-processing, (3) deep bioacoustic signal segmentation[1], and (4) Time-Difference-Of-Arrival (TDOA)-based localization, a large combinatorial variety of different experimental setups is required. Consequently, ORCA-SPY was not only evaluated with respect to different array constellations but also regarding various target signals, including several orca call types combined with boat noise, along with PAMGuard's built-in 2 ms chirp signal. Figure 3 visualizes the nine distinct killer whale call types (N01, N02, N03, N04, N05, N07, N09, N12, and N47), boat noise, and the dampened sine wave of the chirp signal. The call excerpts as well as the noise sample belong to the Call Type Data Corpus (CTDC)[2–4], collected by the OrcaLab[5] and Stephen Ness[6,7].

The excerpt calls contain their own underwater/environmental noise. The actual distance to the recording device, and thus the original signal dampening, is unknown for all these passively captured killer whale samples. As such, the underwater recordings always indicate unknown original SNRs due to overlaying noise, which makes it even harder for the overall system to robustly detect and localize animal positions. Particularly, while facing deteriorating noise conditions and/or additional interfering noise sources (e.g., other boats). Consequently, the ideal and denoised chirp signal (see Figure 3) was used as a reference and indicator while evaluating the best possible performance of ORCA-SPY.

Simulated Localization Scenarios

ORCA-SPY was evaluated on experimental localization scenarios derived on the basis and experience of previous fieldwork expeditions[1]. It incorporates the following practically relevant parametric core concepts and general assumptions (see Figure 4): (1) killer whales traveling around the boat using a radius of 200 m, 400 m, 600 m, 800 m, and 1000 m with respect to the origin (the center of the towed hydrophone array, see Figure 1), (2) orcas vocalizing at 120 equidistant positions ($\Delta 3°$ per call) around the research vessel at various water depths, including 0 m, 100 m, and 200 m, regarding each of the previously mentioned radii, (3) interfering and added boat noise characteristics affecting the emitted killer whale vocalizations, (4) the boat, together with the hydrophone array is not moving during the measurements, and (5) the potential traveling speed of the animals is not taken into account during prediction, which affects the actual position of the orca after localization. PAMGuard's SimSAcq plugin facilitates the generation of additive n-channel sound files (n is the number of hydrophones in the array), by creating several simulated sound sources. Each of the sound sources require a position (angle, distance and depth), sound source level, in relation to the given hydrophone geometry (e.g., an N1 orca call, produced at 200 m distance and 100 m water depth, together with an interfering boat noise at 1,000 m distance and 0 m water depth, both emitted at their chosen sound source levels, utilizing the short array setup). The SimSAcq module appropriately attenuates and shifts the final additive signal with respect to the n-channels hydrophone geometry. In this way, positional real-world datasets can be simulated and produced, leading to varying SNR conditions according to the pre-selected parametric constellations in PAMGuard (position, derived from angle, distance, and depth, as well as the associated sound level of each source). Table 1 and Table 1 summarizes and visualizes the general parametric settings of ORCA-SPY across all experiments. The raw Deep Learning Classifier operates ANIMAL-SPOT[1] across all experiments on a window length of 1.4 s, chosen according to the orca pulsed call time range reported in Ford et al.[8], along with a hop size of 0.7 s, combined with a network confidence of 0.92, and a sampling rate of 44.1 kHz. Moreover, the average duration of about 1.1 s concerning all 9 call type excerpts, visualized in Figure 3, is similar with respect to the chosen ANIMAL-SPOT settings and reported values[8]. To ensure valid segmentation outputs, it is important to choose task-specific and adequate network prediction settings according to the parametric setup during training[1]. The rDLC's segmentation parameters (see Supplementary Table S1), combined with the average vocalization duration of the 9 call types (see Figure 3), typically lead from one to three frame-wise findings per call. Furthermore, PAMGuard's noise-free FFT-data-filter[9], part of the FFT-Engine module (FFT-window-size = 1,024 samples ($\approx$23 ms), FFT-hop-size = 512 samples ($\approx$11.5 ms)), was activated together with the localization in order to counter as many various incoming noise characteristics as possible, highly affecting quality and robustness of ORCA-SPY's localization procedure. Table 1 summarizes and visualizes the chosen parameters.

Table 1 describes all the different experimental localization scenarios examined in this study with respect to the distinct combinatorial compositions based on (1) variety and type of source signals – chirp signal versus noisy orca call types, (2) distance of source origin – 200 m up to 1,000 m, (3) direction source origin – 120 equidistant positions resulting in $\Delta 3°$, (4) water depth source origin – 0 m, 100 m, 200 m, (5) hydrophone array setup – short versus long array, (6) positioning of the hydrophone streamers – elevation/azimuth, and (7) interfering added noise leading to practice-oriented SNR-scales, derived by equation 1. Distinct Sound/Noise source levels, in combination with changing distances and water depths, led to various experimental constellations, resulting in different SNR values, spanning an SNR-scale (see Table 1) utilized to model the following trade-off: (1) simulating SNR scenarios, which lead to significant degradation of ANIMAL-SPOT's detection rates with respect to increasing distances (see Table 1), in order to evaluate ANIMAL-SPOT in worst-case SNR situations, and (2) a sufficient number of valid killer whale segmentations/findings to still guarantee robust evaluation of the localization quality, even under the worst SNR conditions. Interfering added noise, together with increasing ranges (see Table 1), produce critical signal

Parameters	Property	Simulated	Field
Hydrophone	Sensitivity	−170 dB re. 1V/μPa	−170 dB re. 1V/μPa
	Gain (by hydrophone)	61 dB	(0, 8, -2, 2, 0, -1, -1, 0) db
	Dynamic Power Range	90 dB	90 dB
Sound Acquisition	Peak to Peak Voltage Range	2 V	5 v
	Sampling Rate	44,100 Hz	96,000 Hz
ANIMAL-SPOT	Detection Channel	Hydrophone H0	Hydrophone H0
	Window Length	62,000 Samples (1.4 sec.)	134,400 samples (1.4 sec.)
	Hop Size	31,000 Samples (0.7 sec.)	67,200 samples (0.7 sec.)
	Confidence Threshold	0.92	0.85
rDLC	Detection Channel	Hydrophone H0	Hydrophone H0
TDOA	FFT-Window	1,024 Samples	2,048
PAMGuard Noise Free	Median Filter Length	61 (Default)	Disabled
FFT Data Filter[9]	Gaussian Kernel Smoothing	Enabled	Disabled
(Except for Chirp	Threshold	8 dB (Default)	Disabled
Signal in EXP-1)	Average Subtraction	0.02 (Default)	Disabled

Supplementary Table 1. Constant and general parametric setup of ORCA-SPY across all the experiments.The hydrophone gain was adjusted in the field to equalize the output.

constellations, resulting in SNRs which significantly affect ANIMAL-SPOT's detection accuracy, leading to clear segmentation drops starting at about half of the maximal distance, while ending up with detection rates of ≈75 % (SNR = ≈−11 dB) and ≈40 % (SNR = ≈−14 dB) regarding the maximum range (1,000 m distance, 200 m water depth). Consequently, a difference of Δ3 dB at such noisy regions, resulted in a Δ35 % decrease in the detection accuracy, with respect to the maximum distance. The SNR values, according to the specified distance combinations, defined due to their importance in the field (see Table 1), were calculated using the SNR-formula[10]:

$$SNR = (SL - (20 * log_{10}(R_S) + \alpha * R_S)) - (NL - (20 * log10(R_N) + \alpha * R_N))) \quad (1)$$

Given a position-related signal source level SL, a noise level NL, an absorption coefficient α, and R_i indicating the distance to the array center in meters with respect to S (orca) and N (boat). However, the orca calls (see Figure 3) are real-world underwater recordings of the Orchive[5–7] with an unknown noise level. Due to this fact, the SNR can only be calculated under the assumption that the orca call itself has a presumed SNR value above 20 dB and could thereby be considered a "noise-free" signal S, whereas the boat noise corresponds to the actual noise N. Within the pre-selection process of the call types (see Figure 3), attention was paid to ensuring that the original spectral envelope was clearly visible and thus no strong noise interference was present. During all our experiments, the boat noise N was constantly kept at a distance of 1,000 m and 0 m water depth while varying the position of the orca signal S. Moreover, the absorption factor was ignored (and set to zero), because its influence on the overall absorption was less than 0.8 $\frac{dB}{km}$ for a distance of 1,000 m even at 10 kHz frequency. Thus, the transmission loss of Equation 1 was calculated completely bandwidth-independent. Based on the pre-defined distances ranging from 200 m – 1,000 m, combined with pre-assumed water depths of 0 m, 100 m, and 200 m, 15 SNR values (5 distances × 3 depths) can be derived per noise level, adding up to 30 SNRs, providing a merged SNR-based scale with respect to the ANIMAL-SPOT detection rates next to the localization accuracy (see Table 1). To provide an intuitive SNR value (where 0 dB is when the signal and noise amplitudes are similar), peak-to-peak values of the input signal and noise were compared. Note that while it is not recommended to measure peak-to-peak noise levels, the noise in this instance was a defined recording from a boat and thus the ratio of peak-to-peak and root-mean-square (RMS) noise remained constant throughout the simulation (see Figure 3). The RMS-based noise level was at −11.7 dB. Since all the signals (see Figure 3) were amplified to ±15 bit, the peak-to-peak between signal and noise was at 0 dB. However, the RMS signal level is dependent on the call type and the calls range from −15.9 dB (N09) to −10.5 dB (N12) with a median of −13.5 dB (N01). Thus, the signal level SL, together with the noise level NL, would have to be corrected (see Equation 1) by adding the RMS-based signal-/noise-specific values. Consequently, depending on the call type, the peak-to-peak values would have to be corrected by adding −4.2 dB to +1.2 dB to the respective peak-to-peak SNR values visualized in the Supplementary Table S6. Due to previous fieldwork experience occasional occurring artifacts (e.g., clipping) are realistic and can be caused by: (1) large-variety of unexpected multiple and varying levels of noise, (2) invalid human-based and distance-dependent amplifying, and (3) external influences on the hydrophone array as well as improper calibrations. To simulate the occurrence of such artifacts, the experiments were conducted with a pre-amplified gain of 61 dB, leading to a shift

in the dynamic power range to -109 dB re.1V/μPa – -19dB re.1V/μPa. The added noise arriving at the hydrophone can be larger than 109 dB. As such, signal clipping may occur in a few situations. The core motivation is to cover even such situations within the experimental evaluation setup of ORCA-SPY to be as close as possible to real-world scenarios. ANIMAL-SPOT, in combination with the chosen network confidence threshold of 0.92 (see Supplementary Table S1), does not detect an orca call, even in no-noise conditions, if the call falls below ±6 bit at the hydrophone array.

Summary Simulated Scenarios

In total, five different series of simulated experiments (EXP-1 – EXP-5) were conducted while combining constant and modifiable settings shown in main manuscript Table 1 and supplementary Table S1:

In (EXP-1) both towed hydrophone arrays were evaluated together with PAMGuard's built-in 2 ms-long chirp-signal. The Dirac-pulse-like signal was surrounded by perfect silence at a distance of 200 m and depth of 0 m, moving around the research vessel using $\Delta 3°$ intervals. This allows the best possible performance of PAMGuard's TDOA bearing calculator while assuming ideal experimental conditions[11].

(EXP-2): In order to verify the impact of various orca call type structures with respect to localization accuracy, all nine call types were utilized in combination with no added background noise, as well as interfering boat noise. The simulated orca sources were traveling around the boat while producing the respective call types at $\Delta 3°$ intervals, emitted from different distances and water depths. The combining of interfering boat noise results in SNR values between + 3 dB and -4 dB (see Table 1).

(EXP-3) aims to simulate a variety of realistic scenarios by applying the three most frequent "in the wild" occurring killer whale call types (N01, N04, and N09)[6,12] with respect to a large combination of varying distances (200 m to 1,000 m), water depths (0 m, 100 m, 200 m) and added interfering boat noises, at $\Delta 3°$ intervals. These combinations lead to an SNR-scale of +3 dB to -14.2 dB.

(EXP-4): During previous fieldwork expeditions[1] measurements were always performed during moderate driving speed, since driving too slowly caused a sink and/or drift of the hydrophone streamers, which in turn led to inaccurate localization results. Drifting can occur during a change of course or in a strong current at a low speed. However, sinking depends on the speed of the moving boat as well as the buoyancy of the hydrophone array. Both streamers typically sank by about 3.5° at a moderate speed of 2.5 knots according to a depth sensor attached to the streamers, and were less sunken at higher speeds. In general, killer whale localization is disabled in case of too-slow travel speeds. Nevertheless, experimental simulations are mandatory to estimate the impact of drifting and/or sinking array streamers in terms of localization accuracy. During the entire experiment, the assumption was made that the horizontal distance between the two towed-array streamers (23.74 feet, see Figure 1) is constant while drifting to the left or right, as well as in the case of sinking hydrophone streamers. In reality, the horizontal distances between both streamers may deviate, which is strongly dependent on the driving speed. The latter also affects the vertical tension of the streamers, which might be lost as well, leading to twisted streamers. Similar to EXP-3 the N01, N04, and N09 call type were examined at $\Delta 3°$ intervals together with distances of 200 m to 600 m, besides water depths of 0 m, 100 m, and 200 m, and added interfering boat noise causing SNR values of +3 dB to -7 dB. In the case of the long array an array drift of both streamers to the right by an angle of 15° (azimuth $+a = 15°$), and sinking hydrophone streamers leading to an elevation $e = -3°$, $e = -5°$, and $e = -90°$ was evaluated (see Figure 1).

(EXP-5) Whereas EXP-1 to 4 primarily focus on the detection and localization of single isolated killer whale vocalization events, (EXP-5) addresses multiple vocalizing individuals. The most common use for vocalization between killer whales is for intra-pod communication. Only the calls N01, N07, and N47 were utilized for this experiment by random selection. Three different Orcas designated (1) Orca A, which calls the N01 signals every 7 seconds from a 40°bearing at a distance of 200 meters, (2) Orca B, which calls the N07 signal every 8 seconds from a 125 °bearing at a distance of 400 meters and (3) Orca C, which calls the N47 signal every 9 seconds from a 220°bearing at a distance of 200 meters. To synchronize the signals, all calls started at the first second of the recording, of a duration of 115 seconds, 17 N01 calls, 15 N07 calls, and 13 N47 calls were recorded with overlapping windows in accordance to Supplementary Figure S2. In Addition, a second similar simulation was examined with an added interfering noise signal originating from a boat at 305°with an SNR range of +3 to -3 2. Figure 4 graphically represents the general experimental localization scenario, defined according to previous fieldwork experience and recording setups, building the baseline for all previously illustrated experiments (EXP-1 – EXP-5). Besides the specified field-relevant distances (200 m-1000 m) and water depths (0 m, 100 m, 200 m) (see Supplementary Table S1, and Table 1), the constant configurations of ORCA-SPY listed in Table 1 and realistic assumptions regarding the signal source level (orca) and environmental interfering noise level (boat noise) were made. All experiments (EXP-1 – EXP-5) used a constant signal sound source level (SL) of 156 dB re. 1 μPa p-p. Moreover, two noise source levels were applied: (1) NL1 at 167 dB re. 1 μPa p-p within (EXP-2 – EXP-5), and (2) NL2 at 170 dB re. 1 μPa in (EXP-3). The noise source levels are roughly equivalent to a speed boat. Parameterization of these hyperparameters (e.g., other signal/noise levels, distances, etc.) produce different SNRs. Thus, SNR represents the evaluation benchmark for both detection and localization accuracy. Depending on the assumed experimental configurations (see Supplementary Table S1, Table 1), a discrete SNR-scale (see Table 1) can be obtained. By

means of interpolation, a continuous function can be generated with respect to SNR-based detection and localization accuracy in order to be able to map the entire parametric diversity of variants using Equation 1. In Figure 4, besides an interfering boat noise at a constant position, orca individuals were exemplarily visualized at three different positions, together with the chosen noise level and corresponding SNRs (calculated via Equation 1). In addition to Figure 4, Supplementary Figure S1 displays the spectral envelopes of a ±15 bit amplified N09 call type at different SNRs. Next to the corresponding examples visualized in Figure 4, other combinations of distance, depth, source, and noise level are shown. All these factors strongly affect and distort the original signal characteristics. Depending on the chosen settings, orca vocalizations were more or less attenuated, which in turn highly influences orca findings by ANIMAL-SPOT[1], and therefore complicates subsequent localization.

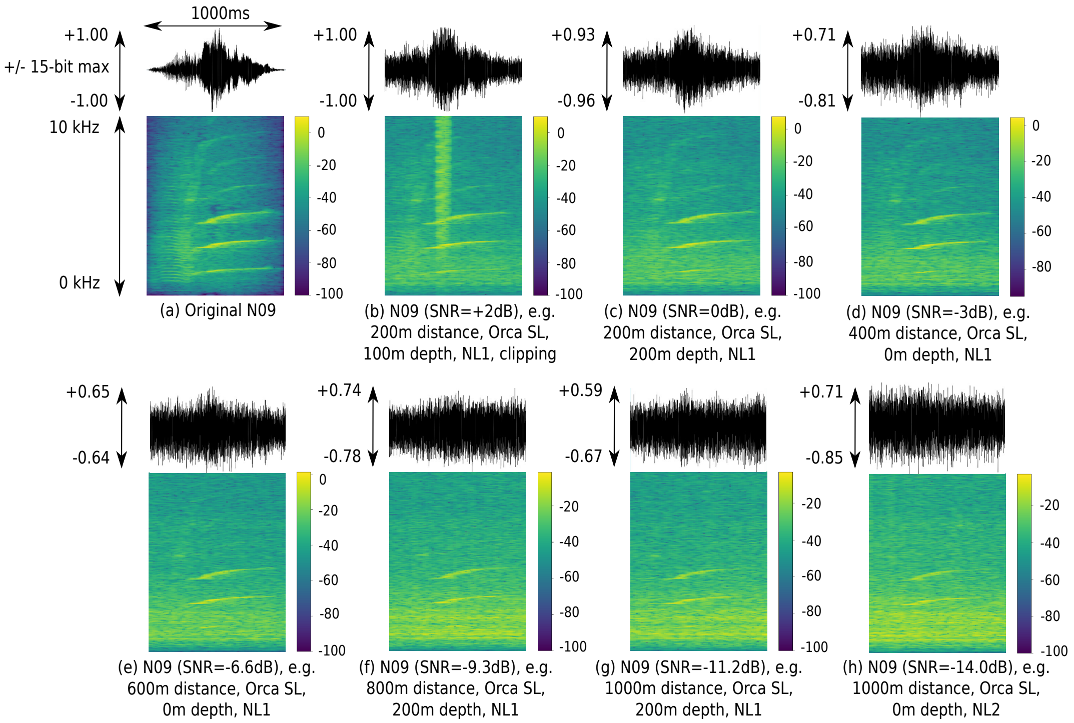

Supplementary Figure 1. Examples of N9 calls at different SNRs, depending on the noise levels, distances, and water depths, which were used in the experiments (b) - (h). The scenarios regarding (e), (f), and (h) are also displayed in Figure 4. SL is the assumed orca signal level of 156 dB, NL1 and NL2 are the different-assumed noise levels of 167 dB and 170 dB with respect to the boat at 1,000 m. (b) is an example for the intentional clipping due to the high gain together with the close distance. Note that all reported SNR values can also result from other parametric constellations regarding distance, water depth, sound, and noise level. These are only example values, derived from the selected real-world and practice-oriented assumptions (see also Table 1).

Results

In the following, the results for each of the five previously mentioned experiments, (EXP-1 – EXP-5) are presented.

(EXP-1) – PAMGuard's Built-In Chirp Signal

The first series of experiments ((EXP-1), see Table 1) was designed to identify a lower boundary regarding the localization error, concerning the hydrophone array setup and the distance between the array and sound source. Thus, only the best constellation was utilized. The short array had a mean error of 0.205°, together with a median of 0.210°, whereas the long array had a mean error of 0.035° and a median of 0.038°. The corresponding distance D between the ground truth and the assumed sound source can be computed via the following equation:

$$D = l * 2 * \sin(\alpha/2) \tag{2}$$

where α is the error in degrees and l is the distance ranging from the hydrophone to the respective sound source. An error of 1° results in a deviation of 3.5 m at 200 m. Supplementary Figure S3 (a) & (b) visualize the results depending on the angle for the

long and short array. The point at 45° on the red line in Supplementary Figure S3 (a) for instance indicates that the position of a chirp, coming from a 200 m distance at an angle of 45° concerning the origin of the long array, is predicted with an error of 0.055°.

(EXP-2) – Different Killer Whale Call Types in Specific Experimental Real-World Scenarios

The second series of experiments ((EXP-2) in Table 1) was designed to get an impression of ORCA-SPY's error in realistic real-world situations and to examine, whether the killer whale call type structure has an influence on the performance and to what extent. According to the combinatorial diversity of (EXP-2), depicted in Table 1, the entire varying parametric setup resulted in nine calls × two radii × 120 angles × three depths × two arrays × two noise constellations equaling a total of 25,920 simulated killer whale call-type activities. As explained in the section Summary Simulated Scenarios, multiple ANIMAL-SPOT findings per call are to be expected. Table 4 displays the number of ANIMAL-SPOT detections with two scenarios: (1) no added environmental noise, and (2) added interfering boat noise, causing SNR values ranging from +3 dB to −4 dB. In total, 59,213 detections, across 25,447 of the 25,920 calls, were received. ANIMAL-SPOT's detection rate was thus 98.2 %. Concerning the 12,960 calls affected by noise (SNR-scale of +3 dB – −4 dB), 24,493 detections were obtained. Overall, 12,503 out of 12,960 calls were correctly identified, leading to a detection rate of 96.5 % within noisy conditions. Regarding the 12,960 vocalization events without the impact of any additional background noise (no added noise condition), ANIMAL-SPOT achieved a detection rate of 99.9 %. The directional tendency (clockwise as positive errors, counterclockwise as negative errors) of all the ≈59,000 detections from (EXP-2) is 0.36° and therefore not showing any indications of a systematic error.
Besides the average localization errors across the different numbers of ANIMAL-SPOT findings (see Supplementary Table S2), a detailed overview with respect to the call type specific triple findings and corresponding localization deviations is given in Supplementary Table S3. Each detection triplet was split into the first, middle, and last detection frame. In case of every call type and prediction frame, the resulting localization errors and respective averages (triple findings) were depicted. Moreover, the call type related average localization divergence with respect to all findings is mentioned. Looking at double findings, the same tendency was observed: The average error of the second finding was higher than the error of the first finding across all calls.
Supplementary Figures S3 (c)-(f) show the localization error depending on the angle for the best (N47), the median (N03), and the worst (N05) call as well as the average of the three calls.

	No Finding	Single Finding	Double Findings	Triple Findings	More Than Three
No added noise (#)	16	8	4,111	8,798	27
Average Error (°)	–	0.01	4.49	6.98	4.05
SNR +3 dB to −4 dB (#)	457	2,050	8,944	1,493	16
Average Error (°)	–	10.30	8.66	10.19	9.74
**SUM** (#)	**473**	**2,058**	**13,055**	**10,291**	**43**
Percentage (%)	1.82	7.94	50.36	39.70	0.16

Supplementary Table 2. Number of ANIMAL-SPOT detections/findings with respect to each killer whale vocalization event (no, single, double, triple, and more than three findings), generated via the combinatorial parametric constellations (in total 25,920) of EXP-2 (see Table 1). The amount of detections/findings, next to the localization error, was calculated across all call types (see Figure 3) within two conditions: (1) no added noise, and (2) interfering boat sound leading to SNR values between +3 dB to −4 dB

(EXP-3) – Most-Frequent Killer Whale Call Types in all Experimental Real-World Scenarios

The third series of experiments (EXP-3 in Table 1) adds even more parametric constellations in distance and noise interference to the three most frequent calls, simulating an entire SNR range of +3 dB to −14.2 dB.
In[6] as well as in[12] these were the call types N01, N04, and N09. Three additional distances and one additional noise interference add up to a total of 32,400 vocalizing killer whale events. ANIMAL-SPOT predicted 63,026 detections across 29,351 call activities based on the 32,400 killer whale signals. Thus, ANIMAL-SPOT's detection ratio was 90.6 %, most likely due to the increased distance and noise interference. However, removing the 2,160 calls within the worst SNR scenario of ≈ −14 dB (see Supplementary Supplementary Table S6) results in 28,342 findings based on the 30,240 simulated orca vocalization events, which in turn yields to 93.7 % detection rate. While only considering the 21,600 vocal activities, with respect to the noisy backgrounds (SNR ranging from +3 dB to −14.2 dB), ANIMAL-SPOT achieved 33,165 detections across 18,558 identified call

Call Type	Triples Found	First Frame	Middle Frame	Last Frame	Average Error of Triples	Average Error of all Detections
N01	1,597	1.80	2.28	6.94	3.67	3.03
N02	1,169	1.96	8.30	13.61	7.96	7.75
N03	724	0.19	0.12	1.27	0.53	4.51
N04	1,361	2.37	2.69	8.81	4.63	3.95
N05	1,442	10.30	10.69	40.06	20.35	19.94
N07	796	2.61	2.81	16.80	7.41	6.56
N09	1,434	7.21	0.37	2.65	3.41	2.58
N12	631	13.19	13.50	20.30	16.67	13.00
N47	1,137	0.73	0.59	3.61	1.64	1.76
∅	**10,291**	**4.37**	**4.32**	**12.80**	**7.23**	**7.01**

Supplementary Table 3. Call type specific localization error regarding the first, second and third detection frame, as well as the average deviation across all three findings for each of the 10,291 identified triplets. In addition, and due to reasons of comparison, the average localization error across all detections/findings is visualized as well (EXP-2).

events, which corresponds to a detection rate of 85.9 %.

Summing up the 18,558 findings of (EXP-3), together with the 12,503 detections of (EXP-2), both predicted within noisy environments lead to a total number of 31,061 orca activity identifications. Hence, the overall detection rate across all simulated killer whale vocalizations, representing the entire SNR-scale of +3 dB to −14.2 dB, caused by the experimental and parametric setup of (EXP-2) and (EXP-3), was 89.9 %. The directional tendency of (EXP-3) is −0.0059° which is negligible with respect to the overall mean error of 3.81° and did not indicate any systematic algorithmic error. Supplementary Supplementary Table S6 displays the results of the third series of experiments (EXP-3 in Table 1). The average localization error of the long hydrophone array is 3.62°, whereas the mean deviation regarding the short hydrophone array is 3.99°, both aligning with the results of (EXP-1). According to Equation 2, the overall mean error of the short array results in a distance between the ground truth and the assumed position of 13.9 m for 200 m, 27.8 m for 400 m, 41.7 m for 600 m, 55.7 m for 800 m, and 69.6 m for 1000 m range to the hydrophone array, whereas the long array results in a distance of 12.6 m for 200 m, 25.3 m for 400 m, 37.9 m for 600 m, 50.5 m for 800 m and 63.1 m for 1000 m to the hydrophone array. Furthermore, Figure 10 presents ANIMAL-SPOT's detection rate in percent, next to the array-specific localization error in degree, both with respect to the SNR in decibels. Therefore, the parametric and experimental scenarios regarding the long and short hydrophone array were combined based on the corresponding SNR-scale, in order to derive a polynomial-based interpolation (spline interpolation), visualizing SNR-driven detection as well as localization results (see Figure 10, Supplementary Table S6). ANIMAL-SPOT's detection rate is ≈100 % at an SNR of −5.2 dB or higher (see Figure 10 (a). The worst possible SNR of −14.2 dB still leads to a ≈43.3 % of detection rate. In Figure 10 (b) the array-specific localization error is visualized across the entire SNR-scale. Supplementary Figure S3 (g) & (h) show the error distribution depending on the sound source angle for the long and short array with respect to the distance and the overall error.

(Exp-4) – Changing Hydrophone Array Position

All previous experiments were conducted under the assumption that the hydrophone array is perfectly parallel to the direction of the boat and on the surface of the water. The purpose of the next series of experiments (EXP-4 in Table 1) was to estimate the additional error if the towed hydrophone array deviates from its ideal position. Table 4 depicts the results for the altered arrays (drifting by 15° to port side $a = 15°$, $e = 0°$, sunken by $e = -3°$, $e = -5°$, and $e = -90°$, all together with $a = 0°$) whereas the results regarding the ideal position are additionally visualized for ease of comparison. Furthermore, Supplementary Figure S3 (i) visualizes the localization error distribution depending on the sound source angle for an azimuth angle of 15° and an elevation angle of −3° and −5°.

(EXP-5) – Multiple Killer Whale Individuals

Unlike the previous experiments where all calls were isolated, (EXP-5) focused on ORCA-SPY behavior with overlapping calls. The main problem with overlapping calls is that the binary classification of a window does not consider how many calls are present, only that at least a call is present. Naturally, the localization algorithm would only focus on the most prominent feature inside the window. Thereby the question of accuracy is somewhat unsuitable, if only the most prominent feature was localized correctly, the remaining calls in said window drop out. As such, the accuracy will only focus on the localized feature.

Long Hydrophone Array										
**Distance**	**200**			**400**			**600**			∅
**Depths**	**0**	**100**	**200**	**0**	**100**	**200**	**0**	**100**	**200**	
**Ideal condition (a = 0°, e = 0°)**										
No added noise	4.20	2.95	2.19	3.70	3.93	2.67	3.52	3.43	3.02	3.29
SNR +3 dB to −7 dB	2.69	1.88	1.69	3.17	2.54	1.92	3.10	3.16	2.35	2.50
**Drifting by 15° to port side (a = 15°, e = 0°)**										
No added noise:	8.33	8.31	7.97	7.88	7.93	7.85	7.52	7.70	7.74	7.91
SNR +3 dB to −7 dB	7.47	7.69	7.62	6.99	7.07	7.00	6.92	6.75	6.88	7.16
**Sunken by 3 degrees (a = 0°, e = −3°)**										
No added noise:	4.08	3.60	3.13	3.86	3.47	3.15	3.41	3.51	3.15	3.48
SNR +3 dB to −7 dB	2.70	2.28	2.59	2.51	2.13	2.10	2.77	3.88	2.71	2.63
**Sunken by 5 degrees (a = 0 °, e = −5°)**										
No added noise:	3.61	3.88	4.18	4.19	3.50	3.77	3.69	3.69	3.75	3.81
SNR +3 dB to −7 dB	3.43	2.69	3.07	3.21	2.99	2.43	3.31	3.21	3.56	3.10
**Sunken by 90 degrees (a = 0°, e = −90°)**										
No added noise:	89.58	90.17	89.79	90.40	89.89	90.08	89.58	89.83	89.36	89.85
SNR +3 dB to −7 dB	91.16	89.59	89.92	89.45	90.16	89.88	89.54	89.55	89.51	89.86

Supplementary Table 4. Overall results of EXP-4: With respect to the long hydrophone array, combined with three possible distances, depths, and no added noise/added noise condition, as well as 120-angle combinations, next to the three most-frequent killer whale call types, the average localization error is reported for: (1) ideal condition (a = 0°, e = 0°), (2) drifting by 15° to port side (a = 15°, e = 0°), (3) sunken by 3° (a = 0°, e = −3°), (4) sunken by 5° (a = 0 °, e = −5°), and (5) sunken by 90° (a = 0°, e = −90°)

The overall error of both simulations is 7.76°. The results of (EXP-5) are displayed in Supplementary Table S5, the overlapping windows and most prominent feature are displayed in Supplementary Figure S2.

**Call**	**No Noise**			**with Noise**		
	**Amount**	**Prominent**	**Error**	**Amount**	**Prominent**	**Error**
Orca A	43	33	5.57	44	44	7.20
Orca B	36	20	3.09	36	10	26.89
Orca C	40	33	6.03	37	30	9.62
∅	**NA**	**86**	**5.17**	**NA**	**84**	**10.41**

Supplementary Table 5. The localization error of the most prominent call inside the overlapping call windows. The overall error of both simulations is 7.76°. The amount of windows a call was present in is displayed in the column Amount, the number of calls, where said call was most prominent inside an overlapping windows and thereby localized, is displayed in column Prominent. The mean error of a call is displayed in column Error.

Long Hydrophone Array																
**Distance**	**200**			**400**			**600**			**800**			**1000**			∅ & #
**Depth**	**0**	**100**	**200**	**0**	**100**	**200**	**0**	**100**	**200**	**0**	**100**	**200**	**0**	**100**	**200**	
No added noise	4.20	2.95	2.19	3.70	3.93	2.67	3.52	3.43	3.02	3.42	3.28	3.25	3.35	3.06	3.27	3.28
# of findings	1,023	1,021	1,022	1,013	1,010	1,008	1,000	997	990	981	980	977	970	967	970	14,929
Added noise (NL1)	2.69	1.88	1.69	3.17	2.54	1.92	3.10	3.16	2.35	3.49	3.27	3.11	4.55	4.03	5.09	3.07
# of findings	844	824	786	747	742	709	668	653	628	524	522	510	412	408	387	9,364
Added noise (NL2)	2.70	1.71	1.45	3.30	2.67	2.27	4.17	4.26	4.78	6.47	4.71	5.50	9.64	7.27	6.92	4.52
# of findings	787	776	716	650	609	610	484	474	476	311	334	325	221	251	220	7,244
overall ∅																**3.62**
overall #																**31,537**
**Short Hydrophone Array**																
**Distance**	**200**			**400**			**600**			**800**			**1000**			∅ & #
**Depth**	**0**	**100**	**200**	**0**	**100**	**200**	**0**	**100**	**200**	**0**	**100**	**200**	**0**	**100**	**200**	
No added noise	4.69	3.90	2.81	4.91	3.91	3.50	4.15	3.93	3.28	3.51	3.81	3.26	3.77	3.60	3.55	3.77
# of findings	1,022	1,021	1,018	1,014	1,013	1,008	997	995	994	979	980	974	971	973	973	14,932
Added noise (NL1)	2.96	2.52	2.34	2.59	2.53	3.15	3.12	2.82	3.10	5.27	4.82	4.11	5.24	5.59	3.81	3.60
# of findings	841	832	801	762	752	725	624	657	632	547	525	524	359	464	388	9,433
Added noise (NL2)	2.44	1.89	2.53	2.68	2.91	3.10	5.94	3.78	4.10	6.88	6.20	5.63	4.89	8.66	7.80	4.63
# of findings	780	774	725	645	643	616	459	466	420	333	302	318	212	220	211	7,124
overall ∅																**3.99**
overall #																**31,489**
**SNR-Scale – Overall Detections**																
No added noise % of detected calls	100	99.7	100	100	100	100	100	99.9	100	100	99.9	99.9	100	99.9	99.9	
Added Noise (NL1) SNR in dB	3	2	0	−3	−3.3	−4	−6.6	−6.7	−7	−9.1	−9.1	−9.3	−11	−11	−11.2	
% of detected calls	100	100	99.9	100	100	100	99.4	100	99.7	91.1	85.6	91.8	75.3	78.1	73.6	
Added Noise (NL2) SNR in dB	0	−1	−3	−6	−6.3	−7	−9.6	−9.7	−10	−12.1	−12.1	−12.3	−14	−14	−14.2	
% of detected calls	100	100	100	99.2	98.9	98.5	83.9	81.5	83.3	68.2	63.8	65.7	47.9	50.6	41.7	
overall ∅																**3.81**
overall #																**63,026**

Supplementary Table 6. Overall results of EXP-3: For each hydrophone array setup, together with every possible distance, depth, no added noise/added noise condition (exemplary NL1/NL2), and 120-angle combination, next to the three most-frequent killer whale call types, the average localization error, the number of findings, as well as the overall average values are reported. Moreover, an SNR-scale is provided, as a result of the different parametric variations, with respect to each experimental constellation, besides the corresponding total amount of identified killer whale vocalizations events.

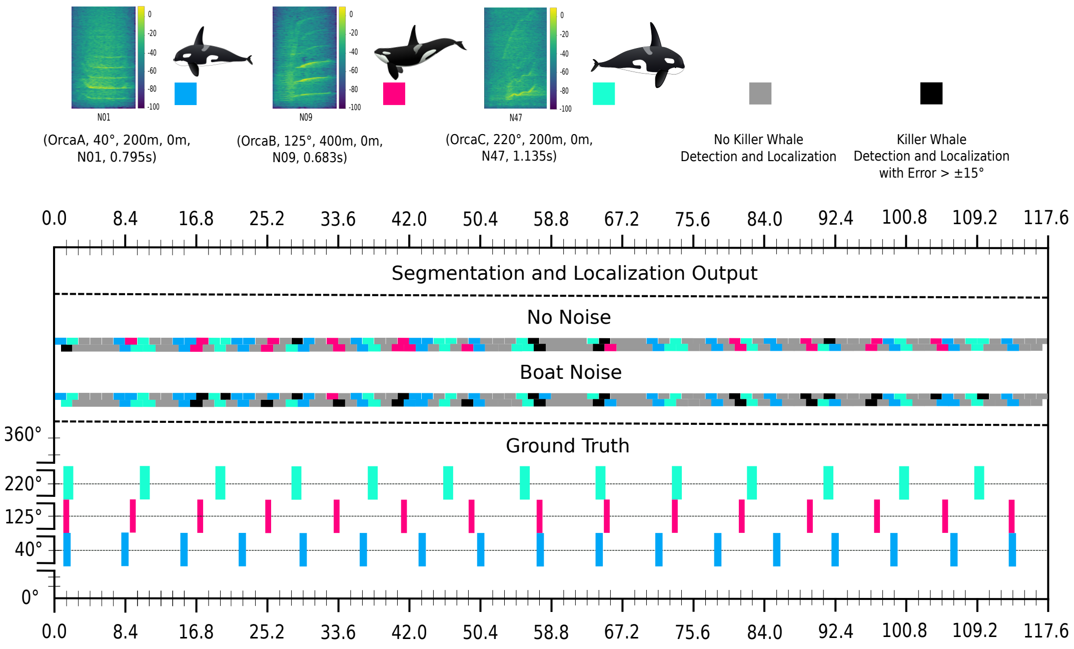

Supplementary Figure 2. The ground truth displays the position of the color coded calls in the simulated environment as well as the overlap. The no noise and boat noise displays the ANIMAL-SPOT windows with their most prominent call.

References

1. Bergler, C. *et al.* ORCA-SPOT: An Automatic Killer Whale Sound Detection Toolkit Using Deep Learning. *Sci. Reports* **9**, DOI: 10.1038/s41598-019-47335-w (2019).
2. Bergler, C. *et al.* Deep Learning for Orca Call Type Identification – A Fully Unsupervised Approach. In *Proceedings of the Annual Conference of the International Speech Communication Association, INTERSPEECH*, 3357–3361, DOI: 10.21437/Interspeech.2019-1857 (2019).
3. Bergler, C. *et al.* Deep Representation Learning for Orca Call Type Classification. In *Text, Speech, and Dialogue, 22nd International Conference, TSD 2019, Ljubljana, Slovenia, September 11–13, 2019, Proceedings*, vol. 11697 LNAI, 274–286, DOI: 10.1007/978-3-030-27947-9{_}23 (Springer Verlag, 2019).
4. Bergler, C. *et al.* ORCA-CLEAN: A Deep Denoising Toolkit for Killer Whale Communication. In *Proc. Interspeech 2020*, 1136–1140, DOI: 10.21437/Interspeech.2020-1316 (2020).
5. ORCALAB. ORCALAB - A whale research station on Hanson Island. https://orcalab.org/ (May 2021).
6. Ness, S. *The Orchive : A system for semi-automatic annotation and analysis of a large collection of bioacoustic recordings*. Ph.D. thesis, Department of Computer Science, University of Victoria, 3800 Finnerty Road, Victoria, British Columbia, Canada, V8P 5C2 (2013).
7. Ness, S. Orchive. http://orchive.cs.uvic.ca/ (May 2021).
8. Ford, J. K. B. Acoustic behaviour of resident killer whales (*Orcinus orca*) off Vancouver Island, British Columbia. *Can. J. Zool.* **67**, 727–745 (1989).
9. Oswald, M. https://sourceforge.net/p/pamguard (2021).
10. Urick, R. *Principles of Underwater Sound* (McGraw-Hill, 1983).
11. Gillespie, D., Palmer, L., MacAulay, J., Sparling, C. & Hastie, G. Passive acoustic methods for tracking the 3D movements of small cetaceans around marine structures. *PLoS One* **15**, DOI: 10.1371/journal.pone.0229058 (2020). This research was funded through a research grant from the Scottish Government as part of the Marine Mammal Scientific Support Program MMSS/002/15.
12. Bergler, C. *et al.* ORCA-SLANG: An Automatic Multi-Stage Semi-Supervised Deep Learning Framework for Large-Scale Killer Whale Call Type Identification. In *Proc. Interspeech 2021*, 2396–2400, DOI: 10.21437/Interspeech.2021-616 (2021).

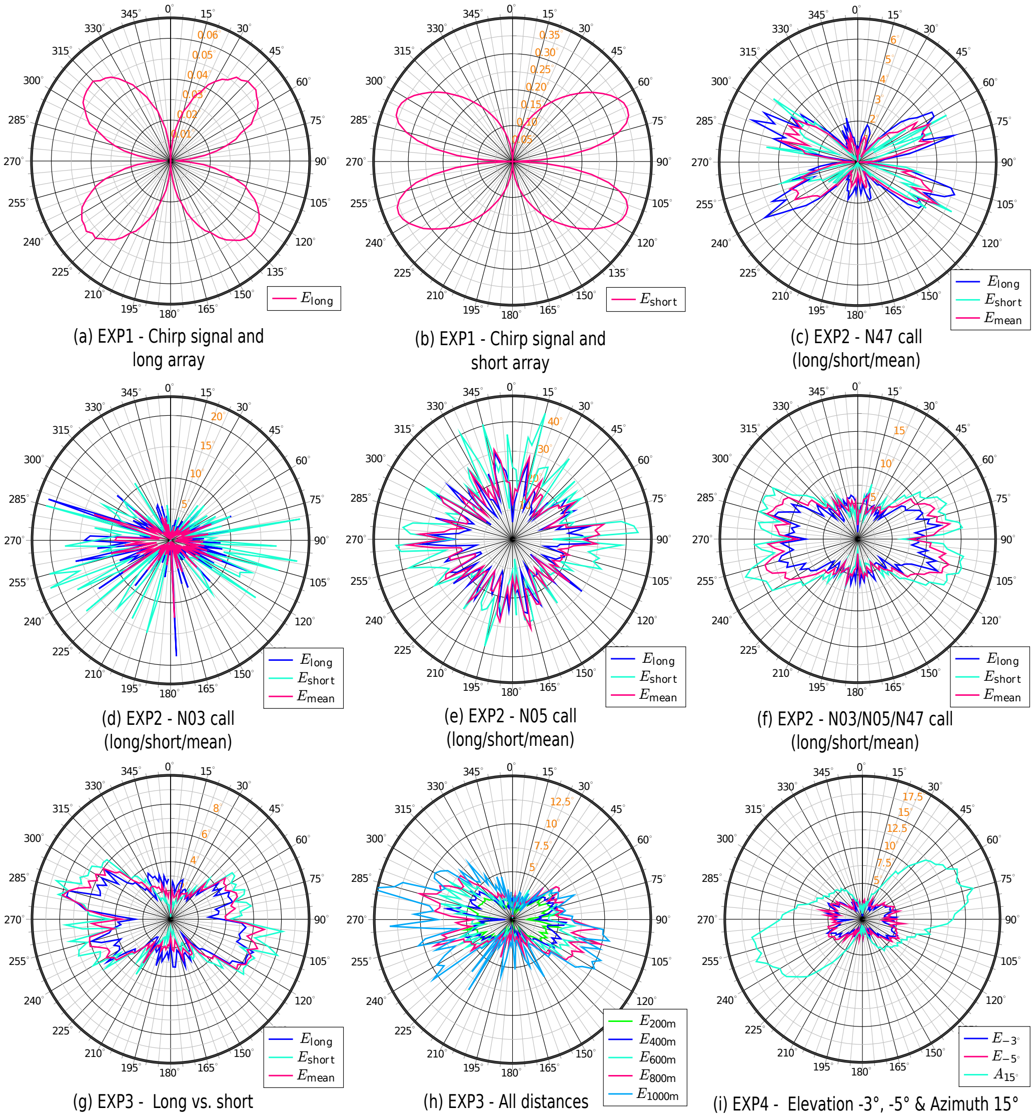

(a) EXP1 - Chirp signal and long array
(b) EXP1 - Chirp signal and short array
(c) EXP2 - N47 call (long/short/mean)
(d) EXP2 - N03 call (long/short/mean)
(e) EXP2 - N05 call (long/short/mean)
(f) EXP2 - N03/N05/N47 call (long/short/mean)
(g) EXP3 - Long vs. short (mean of all distances)
(h) EXP3 - All distances (mean of long/short array)
(i) EXP4 - Elevation -3°, -5° & Azimuth 15° (mean of long/short array)

Supplementary Figure 3. Best possible performance of the long and short array and experimental results for different orca calls and conditions.
(a), (b): Error depending on the angle w.r.t. the center of the array for a chirp call under no-noise condition (see results EXP-1).
(c) – (f): Error depending on the angle w.r.t. the center of the array for the best (N47), the median (N03), and the worst (N05) of the nine calls and the average of these calls (see results EXP-2).
(g), (h): Error depending on the angle w.r.t. the center of the array for the most frequent calls (N01, N04, N09). (g) shows the results for the long and short array and for the overall average. (h) shows the results depending on the distance of the calls (see results EXP-3).
(i) Error depending on the angle w.r.t. the center of the array if the array is not in the assumed position. The results for $e = -90°$ are not displayed (see results EXP-4).

GPS Bearing	Distance to Array	Mean Error	Median Error	Amount of Locs	Amount below 15°	Percentage below 15°
2.10	67.74	21.38	19.83	38	16	42.11
3.87	76.54	39.03	33.06	464	92	19.83
15.74	65.74	48.70	30.18	139	15	10.79
21.28	40.33	53.65	54.34	154	12	7.79
37.93	37.34	42.78	35.59	138	27	19.56
47.58	68.25	29.76	25.76	77	17	22.07
75.41	68.38	23.12	16.89	73	30	41.10
77.81	43.19	21.39	13.63	141	92	65.25
79.45	65.61	21.80	13.84	129	72	55.81
106.31	55.19	26.79	5.88	67	47	70.15
106.86	43.55	35.35	21.10	137	53	38.69
110.77	70.86	18.83	7.06	152	110	72.37
124.29	56.19	16.19	4.29	63	49	77.78
135.07	47.45	31.60	22.83	159	58	36.48
137.05	47.94	28.41	19.48	131	50	38.17
162.48	86.36	19.24	6.69	59	49	83.05
168.44	49.34	10.25	5.27	169	153	90.53
169.08	65.33	43.35	27.66	98	28	28.57
188.33	43.99	31.20	20.11	55	25	45.45
192.58	41.35	43.56	24.14	211	82	38.86
203.46	20.27	17.24	13.30	174	107	61.49
219.00	27.08	14.55	10.72	25	15	60.00
239.95	29.83	20.20	16.41	187	83	44.39
247.49	56.96	27.45	21.92	258	59	22.87
285.64	77.24	11.67	7.80	181	134	74.03
318.08	95.29	18.66	4.67	30	23	76.67
319.84	106.13	47.47	26.41	21	9	42.86
323.92	52.50	33.21	13.28	101	54	53.47
343.97	68.53	17.97	8.36	116	123	74.10
345.95	45.80	15.77	9.59	51	35	68.63
346.68	92.73	37.39	21.11	41	12	29.27
∅	55.31	29.19	17.54	3889	2883	71.13

Supplementary Table 7. Summary of the results of the lake Stechlin expedition data. The GPS-bearing is the ground truth of the real data set, the bearing from where the expedition vessel was sending the signals via underwater loudspeakers. The distance to array is the distance from the expedition vessel to the array center in meter. The mean error is the absolute mean error from the localization to the GPS-bearing. The median error is the absolute median of all localization errors. The amount of locs is the amount of localizations found at this GPS-position. The amount below 15 °is the amount of localization absolute errors below 15. The percentage below 15 °is the percentage of calls with an absolute error below 15 °with respect to all calls.
